# Biomarkers of intake for tropical fruits

**DOI:** 10.1186/s12263-020-00670-4

**Published:** 2020-06-19

**Authors:** N. Vázquez-Manjarrez, M. Ulaszewska, M. Garcia-Aloy, F. Mattivi, G. Praticò, L. O. Dragsted, C. Manach

**Affiliations:** 1 Human Nutrition Unit, Université Clermont Auvergne, INRAE, F-63000 Clermont-Ferrand, France; 2 Department of Nutrition, Exercise and Sports, University of Copenhagen, Copenhagen, Denmark; 3 Dirección de Nutrición, Instituto Nacional de Ciencias Médicas y Nutrición Salvador Zubirán, Mexico City, Mexico; 4 Research and Innovation Centre Food Quality and Nutrition, Fondazione Edmund Mach, Via Mach 1, 38010 San Michele all’Adige, Italy; 5 Biomarkers and Nutrimetabolomic Laboratory, Department of Nutrition, Food Sciences and Gastronomy, XaRTA, INSA, Faculty of Pharmacy and Food Sciences, Campus Torribera, University of Barcelona, Barcelona, Spain; 6 CIBER de Fragilidad y Envejecimiento Saludable (CIBERFES), Instituto de Salud Carlos III, Barcelona, Spain; 7 Department of Cellular, Computational and Integrative Biology, CIBIO, University of Trento, San Michele all’Adige, Italy

**Keywords:** Banana, Watermelon, Avocado, Pomegranate, Tropical fruit, Biomarkers, Intake

## Abstract

Consumption of fruit and vegetable is a key component of a healthy and sustainable diet. However, their accurate dietary assessment remains a challenge. Due to errors in self-reporting methods, the available dietary information is usually biased. Biomarkers of intake constitute objective tools to better reflect the usual or recent consumption of different foods, including fruits and vegetables. Partners of The Food Biomarker Alliance (FoodBall) Project have undertaken the task of reviewing the available literature on putative biomarkers of tropical fruit intake. The identified candidate biomarkers were subject to validation evaluation using eight biological and chemical criteria. This publication presents the current knowledge on intake biomarkers for 17 tropical fruits including banana, mango, and avocado as the most widely consumed ones. Candidate biomarkers were found only for banana, avocado, and watermelon. An array of banana-derived metabolites has been reported in human biofluids, among which 5-hydroxyindole-acetic acid, dopamine sulfate, methoxyeugenol glucuronide, salsolinol sulfate, 6-hydroxy-1-methyl-1,2,3,4-tetrahydro-β-carboline-sulfate, and other catecholamine metabolites. Their validation is still at an early stage, with insufficient data on dose-response relationship. Perseitol and mannoheptulose have recently been reported as candidate biomarkers for avocado intake, while the amino acid citrulline has been associated with watermelon intake. Additionally, the examination of food composition data revealed some highly specific phytochemicals, which metabolites after absorption may be further studied as putative BFI for one or several tropical fruits. To make the field move forward, untargeted metabolomics, as a data-driven explorative approach, will have to be applied in both intervention and observational studies to discover putative BFIs, while their full validation and the establishment of dose-response calibration curves will require quantification methods at a later stage.

## Background

More than 800 tropical fruits have been described, but only a small number are widely consumed [1]. Geographically, tropical fruits have their origin in the tropics of Cancer and Capricorn in the north and south of the equator covering most of the tropical and subtropical areas of Asia, Africa, Central America, South America, the Caribbean, and Oceania. In comparison to the temperate fruits such as apple, pears, and berries, tropical fruits are gaining terrain in global production and trade due to a growing demand from consumers. As an example, the production of avocados in the top producing country, Mexico, rose 1900% between 1961 and 2017 [2]. Moreover, mango availability was reported to increase from 1 kg per capita in 2007 to 1.8 kg per capita in 2017 in the USA, and from 0.4 kg to 0.7 kg in the European Union [3].

Several tropical fruits have been studied for their effect in human health due to their content in specific bioactive compounds. Of these, pomegranate has received much attention in recent years. A recent meta-analysis of 8 randomized controlled trials (RCT) (*n* = 574) showed that the intake of pomegranate juice reduces systolic (− 4.96, 95% CI − 7.67 to − 2.25 mmHg, *P* < 0.001) and diastolic blood pressure (− 2.01, 95% CI − 3.71 to− 0.31, *P* = 0.021) [4]. The effects of pomegranate on human health, including LDL-cholesterol, triglycerides, and glucose reduction, have been ascribed to its content in ellagitannins and to urolithins, their gut microbiota metabolites [5, 6].

Unlike pomegranate, information regarding the health effects of other tropical fruits, including the widely consumed banana, is remarkably scarce. To tackle this, well-conducted human studies with randomized controlled designs and prospective cohort studies with accurate dietary assessment will be needed.

Food Frequency questionnaires (FFQs) are widely used to assess fruit intake, but they have several disadvantages. It has been shown that the intake of fruits and vegetables (F&V) is often over-reported when assessed by food frequency questionnaires, due to social desirability bias [7]. Moreover, dietary questionnaires rarely evaluate the fruit consumption down to specific foods and varieties, and even in detailed FFQs, not much attention is given to tropical fruits, because of their diversity and low average intake in most studied populations compared to other fruits. In Western countries, except for banana and avocado, tropical fruits are often not considered in the FFQs or they are integrated in a more or less heterogeneous food group. For example, watermelon and melon are constantly listed together [8, 9] hindering the assessment of their respective intakes. In some producing countries such as Singapore, Mexico, and Brazil, FFQs consider tropical fruits including guava, kiwi, papaya, dragonfruit, watermelon, dates, and persimmon [10–12], although in an inconsistent manner. One could argue that the well-designed dietary instruments may suffice to obtain accurate intake information of tropical fruits. However, self-reporting methods will always be subject to imprecision associated to recall errors, difficulty to assess portion size, and social desirability bias. Furthermore, listing all tropical fruit individually would significantly expand the questionnaires and increase participant burden. The use of dietary biomarkers, whose associated errors are independent from that of questionnaire instruments, has the potential to assist in accurately determining dietary intake, and hence better unravel the associations between diet and human health.

BFIs originate from compounds that are specific for a given food or food group, and become bioavailable after their consumption [13]. Plasma vitamin C and carotenoids increase after tropical fruit intake and are biomarkers commonly used to assess the total intake of F&V [14]. However, their use is not without limitations. The bioavailability of carotenoids fluctuates according to the alongside diet or food preparation, e.g., simultaneous intake of fat enhances their absorption [15, 16]. Furthermore, plasma vitamin C concentration exhibits saturation kinetics, meaning that its collinearity with dietary intake stops at a certain point, especially in well-nourished subjects where baseline concentrations are observed at ~ 60 μmol/L [17]. Plasma vitamin C is further challenged by several external factors including pre-analytical conditions such as sample handling, temperature, and conditions of storage [18].

The Food Biomarkers Alliance (FoodBAll JPI-project; www.foodmetabolome.org), in an effort to collate the most comprehensive panel of BFIs as possible for over 140 commonly consumed foods, has conducted a series of reviews of the literature according to the guidelines established by the consortium [19]. The obtained BFIs are evaluated according to eight defined criteria to qualify their applicability as BFIs in further nutritional and health-related research [20]. The vision is to develop analytical methods that could quantify in a cost-effective way a battery of dietary biomarkers to validate or complement dietary questionnaires. Within the FoodBall initiative, the objectives of the present work are as follows: (1) retrieve from an extensive literature search the compounds currently used as BFIs for tropical fruits and those which are specific enough to represent putative BFIs and (2) outline the available knowledge and provide the current level of validation of the collated candidate BFIs for tropical fruits.

## Methods

### Selection of food groups

For the present review, 16 tropical fruits including the most consumed in the world according to FAO production and trading data were selected [3]. This review assesses the following fruits: dessert banana (*Musa acuminata)*, mango (*Mangifera indica*), pineapple (*Ananas comosus*), papaya (*Carica papaya*), avocado (*Persea Americana*), pomegranate (*Punica granatum*), kiwifruit (*Actinidia*), lychee (*Litchi chinensis*), persimmon (*Diasporos kaki*), guava (*Psidium guava*), passion fruit (*Passiflora edulis*), acerola (*Malpighia emarginata*), dragon fruit (*Hylocereus undatus*), coconut (*Cocos nucifera*), watermelon (*Citrullus vulgaris*), muskmelon (*Cucumis melo*), and date (*Phoenix dactylifera*).

### Search for relevant BFIs research papers

An extensive literature search was carried out to collect all available information on the already used or new putative BFIs for the selected fruits. The BFIRev protocol (Food Intake Biomarker Reviews) elaborated with the guidance of the PRISMA statement (Preferred Reporting Items for Systematic reviews and Meta-Analyses) and described in Pratico et. al. [19] was followed. Briefly, a primary search was performed in the three literature search databases, Scopus, PubMed central, and Web of Science, with the name of the specific fruit and its botanical genus, i.e., (Banana OR Musa*) OR (Mango OR Mangifera* OR Pineapple OR Ananas* OR Papaya OR Carica* OR Avocado OR Persea* OR Pomegranate OR Punica* OR Kiwi* OR Actinidia* OR Lychee OR Lichee OR Litchi* OR Persimmon OR Diospyros* OR Guava OR Psidium* OR Passion fruit OR Passiflora* OR Acerola OR Malpighia* OR Pitaya OR Pitahaya OR Dragon fruit OR Hylocereus* OR Coconut OR Cocus* OR Watermelon OR Citrullus* OR Muskmelon OR Melon OR Cantaloupe OR Cucumis* OR Phœnix dactylifera) along with the common keywords: AND (urine OR plasma OR serum OR excretion OR blood) AND (Human* OR men OR women OR patient* OR volunteer* OR participant*) AND (Biomarker* OR marker* OR metabolite* OR Biokinetics OR Biotransformation OR Pharmacokinetics OR bioavailability OR ADME) AND (Intake OR meal OR diet OR ingestion OR administration OR consumption OR eating OR drink*). Keywords were used in the fields [Topic], [All fields], and [Article Title/Abstract/Keywords] for Web of Science, PubMed, and Scopus, respectively. All searches were carried out in March 2016 and updated in January 2019. A last search in April 2020 resulted in no new putative biomarkers. Only papers in the English language were considered eligible, and no restriction on the date of publication was applied. Articles showing results of human intervention studies (randomized controlled trials, single-dose, short-term or long-term studies) or observational studies (cohort, case-control, cross-sectional studies) were considered eligible. After removal of duplicates, a first selection of papers was performed according to the relevance of the abstract and title. Full texts were obtained for the selected articles and further assessed for eligibility according to their relevance for the identification of BFIs for all tropical fruits. Some of the publications found in the reference list of the selected articles were also included at this stage. Furthermore, for those tropical fruits for which no information in human studies was available, we carried out an additional search in food composition databases including, Dictionary of Food Compounds, FooDB and KNApSAck [21–23], and used the text-mining tools, Polysearch2 [24] and Coremine [25] medical online database, to identify specific compounds for each fruit that may be further investigated as putative BFIs [26].

### Characterization of candidate BFIs

For each putative biomarker identified, a secondary search allowed to retrieve relevant information to assess its specificity, pharmacokinetics, dose-response relationship, robustness, and reliability, as well as the quality of its method(s) of analysis, in order to qualify its use as BFI according to the validating scheme established by Dragsted et al. [20].

The name of the putative biomarkers and their synonyms were queried in the literature search databases along with AND (biomarker* OR marker* OR metabolite* OR biokinetics OR biotransformation OR pharmacokinetics OR bioavailability OR ADME). Additionally, the compounds were searched manually in the online databases HMDB [27], FooDB [22], Phenol-Explorer [28], Dictionary of Food Compounds [21], Duke’s phytochemical and ethnobotanical databases [29], eBASIS [30], KNApSack [23], and PhytoHub [31] to determine all the possible dietary or metabolic origins of the candidate BFIs.

The list of candidate BFIs was reviewed and agreed upon all authors.

### Application of validation criteria

According to the method of Dragsted et al. [20], a validation assessment procedure was applied on the candidate BFIs to assess their current status of validation and identify the missing information for full validation. The validation scheme is based on eight questions that encompass biological and chemical aspects: plausibility, dose-response, time-response, robustness, reliability, stability, analytical performance, and reproducibility (Table S1).

## Results and discussion

**Fig. 1:**
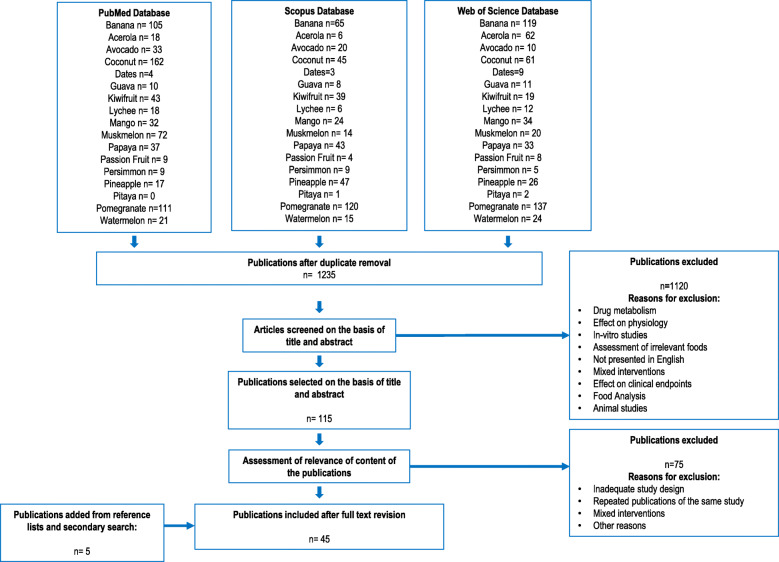
Flow diagram of study selection according to the BFIRev procedure

**Fig. 2:**
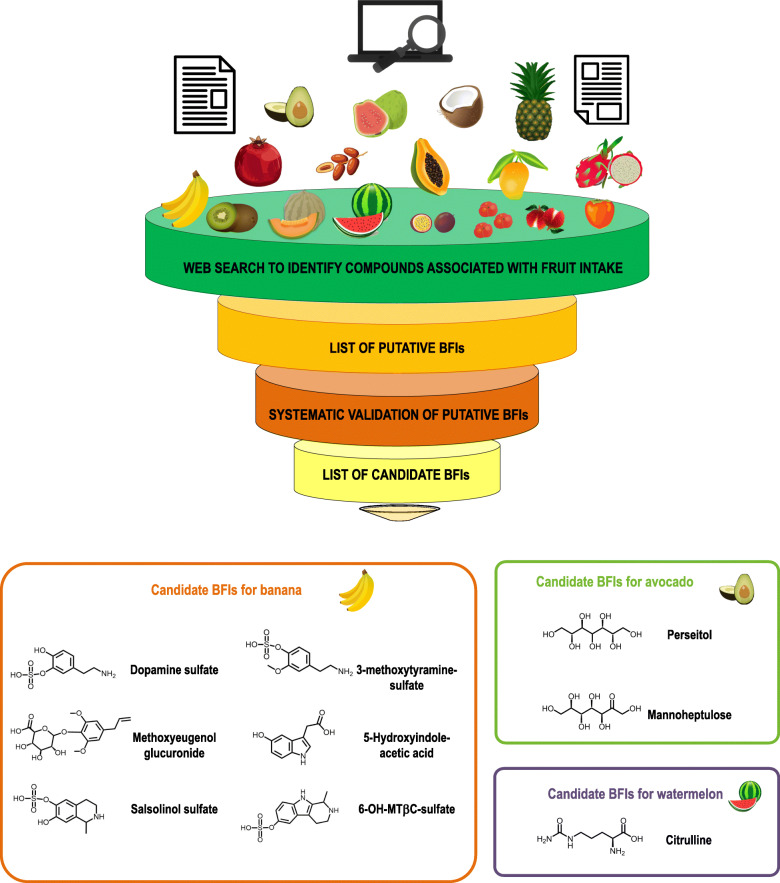
Overview of the literature evaluation process of the candidate biomarkers for banana and avocado, and the candidate biomarker for watermelon

A flowchart indicating the literature search and the review process is shown in Fig. 1. After removal of duplicates, the literature search yielded 1235 publications for the tropical fruits. Of these, 40 articles reported relevant information on putative biomarkers of intake. Five additional articles were selected from the reference list of the assessed publications or from the secondary search. A summary of the retained literature and the list of the specific candidate BFIs for tropical fruits are presented in Table 1 and Fig. 2. The level of validation of the candidate BFIs is shown in Table 2. For an exhaustive presentation of the results, a full list of all retained and non-retained compounds is given in Table S2, with the main reasons for inclusion or exclusion and the corresponding references.

**Table 1:** List of publications reporting candidate biomarkers for banana, watermelon, and avocado consumption

Dietary factor	Study design	Number of subjects	Analytical method	Sample type	Discriminating metabolites/candidate biomarkers	Primary reference
Banana (4 fresh fruits)	Human intervention study Habitual diet supplemented with banana and other fruits.	8 (5 women, 3 men)	Radio enzymatic assay	Urine (24 h collection)	5-Hydroxindole acetic acid	[32]
Banana (300–500 g of fresh fruit)	Controlled study One-day of intervention: (1) banana pulp, (2) control (oral dose of serotonin)	3 (3 men)	Paper chromatography with a fluorometric detection	Urine (24 h collection in 6 time points)	5-Hydroxindole acetic acid	[33]
Banana (450 g of fresh fruit)	Randomized controlled meal study with four intervention days: (1) bread as control, (2) banana, (3) alcohol, and (4) alcohol + banana	10 (7 women, 3 men)	HPLC	Urine (24 h collection in 9 time points)	5-Hydroxindole acetic acid	[34]
Banana (325–468 g of fresh fruit)	Acute intervention One-day run-in period followed by one day of intervention by adding three to four bananas to conventional diet; after 24 h subjects resumed to regular diet.	5 (3 women, 2 men)	GC-MS HPLC-ECD	Urine (3 days collection during daytime in 2–3 h intervals)	5-Hydroxindole acetic acid	[35]
Banana (4 fresh fruits)	Randomized controlled study Six-day intervention: (1) control diet, (2) 7 g vanilla plus control diet, (3) banana plus control diet	12 (6 men, 6 women)	HPLC-ECD	Urine (24 h collection)	5-Hydroxindole acetic acid	[36]
Banana (50 g of fresh fruit)	Meal intervention Two loadings separated by 12 h	2 (1 woman,1 man)	HPLC	Urine (1, 3, and 6 h after intake and first void of urine after the second period of intake)	5-Hydroxyindole acetic acid	[37]
Banana (200 g of fresh fruit)	Control, crossover intervention study 13 days of intervention 1 day per food tested: (1) tested foods: 200 g of banana, pineapple, tomato, kiwi, orange, 30 g walnut, 1 g vanilla beans, 100 g papaya, 200 g apple, 100 g spinach, 100 g cucumber, and 15 g coffee beans; (2) a cup of milk and 70 g of white bread as control	3 (all women)	HPLC-ECD	Urine (random urine collection 3 h after intake)	5-Hydroxindole acetic acid	[38]
Banana (200 g of fresh fruit)	Single-dose food intervention	3 (2 women, 1 man)	HPLC-ECD	Urine (collected every hour from 2 to 7 h after the intake )	5-Hydroxyindole acetic acid	[38]
Banana (200 g once a day at different times)	Meal intervention over five consecutive days First day dietary restriction, second day 200 g of banana at 12:00 h, on the third day 200 g of banana at 16:00 h and on the fourth day 200 g of banana at 20:00 h.	9 (2 men, 7 women)	HPLC-ECD	Urine (first and second urine after banana intake over 5 consecutive days)	5-Hydroxyindole acetic acid	[38]
Banana (12 fresh fruits)	Controlled intervention One day of intervention per food tested: (1) “plant free” control diet, (2) control diet plus tested food.	2 (1 woman,1 man)	Two-dimensional paper chromatography Ion exchange column chromatography	Urine (12 h and 24 h collection)	5-Hydroxyindole acetic acid	[39]
Banana (fresh fruit given once a day)	One-day study with three banana meal interventions within a single day: (1) 205 g of banana divided in three meals, (2) 475 g of banana divided in three meals, (3) 725 g of banana divided in three meals.	2 (healthy volunteers	Paper chromatography fluorometric determination	Urine (24 h urine collections)	5-Hydroxyindole acetic acid	[40]
Banana (one and two fresh fruits)	Acute study with two interventions: (1) one banana with lunch, (2) two bananas with lunch	6 (sex not specified)	Paper chromatography fluorometric determination	Urine (2 h after banana intake and 4 h after banana intake)	5-Hydroxyindole acetic acid	[40]
Banana (> 350 g of banana)	Meal intervention preceded by 3 days of run in period with low serotonin containing foods. Tested foods (group A and B): 297–362 g pineapple, 335 g kiwi, 102 g walnuts, and 385–359 g of banana	35 (group A = 12, 8 women and 4 men; group B = 23, all women)	HILIC-QTRAP-MS/MS	Serum (group A : 0 h, 0–2 h, 2–4 h, 4–6 h, 24 h, 48 h, 72 h after intake; group B: 0 h, 24 h, 48 h, and 72 h after intake)	5-Hydroxindole acetic acid	[41]
Banana (freeze-dried banana)	Randomized, crossover, controlled study Three days of intervention: (1) control, (2) pear 0.4 g/kg first loading followed by 0.6 g/kg/h during bicycling challenge, (3) banana first loading 0.4 g/kg followed by 0.6 g/kg/h during bicycling challenge	20 (all men)	UHPLC-MS/MS	Plasma (fasting sample, 1.5 h after physical challenge and 21 h post exercise)	5-Hydroxyindole acetic acid Dopamine sulfate	[42]
Banana (one banana as part of a standard breakfast)	Parallel meal study with two treatments: (1) ethanol infusion, (2) banana containing breakfast followed by ethanol infusion 1 h after intake	11 (all men)	HPLC-ESI-MS/MS	Plasma (group 1: 0 h, 15 min,45 min; group 2: 0 h, 0–1 h after banana intake then 15 min, 30 min, 45 min after ethanol infusion)	Dopamine (R + S) Salsolinol	[43]
Banana	Crossover controlled study on a single intervention day: (1) control, (2) banana (~ 6–7 fruits)	14 (all men)	GC-MS	Plasma (1 h pre-exercise, immediately after exercise, 1 h post exercise)	Dopamine	[44]
Banana (100 g of dried banana)	Acute intervention One day of intervention with banana	6 (all men)	HPLC-ECD	Urine (24 h collections in two fractions per day 8 am to 5 pm and 5 pm to 9 am; 2 days of sampling: day before and day after banana intake)	(R + S) Salsolinol	[45]
Banana (5–20 bananas a day)	Case report	1 (woman)	Not reported	Whole blood (1 collection per month of follow up)	Dopamine	[46]
Banana (freeze-dried banana)	Randomized controlled crossover study with four interventions: (1) First loading of 5 mL/kg of water as control followed by 3 mL/kg/15 min until end of 75 km cycling challenge, (2) first loading of 0.4 g/kg of Cavendish banana followed by 0.2 g/kg every 15 min until end of 75 km bicycling challenge 3) 0.4 g/kg of mini yellow banana followed by 0.2 g/kg/15 min until end of 75 km bicycling, (4) 6% sugar beverage every 15 min until end of 75 km bicycling	20 (14 men, 6 women)	UHPLC-MS/MS	Serum	5-hydroxyindole-acetic acid Dopamine-3-O-sulfate Dopamine-4-sulfate	[47]
Banana FFQ, 3.01 g (0.98–3.01 g)^a^	Cross-sectional study	1369 (all postmenopausal women)	UHPLC-MS/MS	Serum (non-fasting, one collection)	5-Hydroxyindole acetic acid Dopamine-3-O-sulfate Dopamine-4-sulfate 3-Methoxytyramine-sulfate 5-Hydroxyindole acetic acid	[48]
Banana (240 g of fresh fruit)	Randomized controlled study Two days run-in period with restricted diet; three intervention periods with at least 3 days of wash-out period between, (1) 250 mL control drink, (2) 240 g of banana + 150 mL of control drink, (3) 300 g of tomato + 12 g of sunflower oils + 150 mL of control drink	12 (6 men,6 women)	UHPLC-QTOF-MS	Urine (24 h collection)	Methoxyeugenol-glucuronide + Dopamine sulfate Methoxyeugenol-glucuronide + Dopamine sulfate+ Salsolinol sulfate Methoxyeugenol-glucuronide + Dopamine sulfate+6-OH-MTβC sulfate +2-isopropylmalic acid Methoxyeugenol-glucuronide + Dopamine sulfate +Salsolinol sulfate+ Xanthurenic acid+6-OH-MTβC sulfate	[49]
			GCxGC-MS	Urine (24 h collection in seven time points)	Dopamine Methoxyeugenol Salsolinol	
Banana (24 h recalls, (1) high consumers 176 g (126–378 g), (2) low consumers 87.7 g (47.3–94.5 g), (3) non-consumers)^b^	Cross-sectional study	78 (39 men, 39women)	UHPLC-QTOF-MS	Urine (24 h collection)	Methoxyeugenol-glucuronide + Dopamine sulfate Methoxyeugenol-glucuronide + Dopamine sulfate+ Salsolinol sulfate Methoxyeugenol-glucuronide + Dopamine sulfate+6-OH-MTβC sulfate +2-isopropylmalic acid Methoxyeugenol-glucuronide + Dopamine sulfate + Salsolinol sulfate+ Xanthurenic acid+6-OH-MTβC sulfate	[49]
Watermelon (3.3 kg wet weight of fruit)	Human study intervention	6 (sex not specified)	Ion exchange chromatography	Plasma	Citrulline	[50]
Watermelon juice (300 mL)	Double blind crossover-controlled study; 16-day supplementations with seven to ten days washout period; (1) control, (2) 300 mL of watermelon juice, (3) 300 mL of apple concentrate juice	8 (all men)	Fluorescence-detection HPLC	Plasma (five collections following different bicycling challenges)	Citrulline	[51]
Watermelon puree (980 mL/day)	Randomized placebo-controlled crossover study; two interventions: (1) 6% carbohydrate beverage as control, (2) 980 mL/day of watermelon puree for 2 weeks. On the day of physical challenge subjects ingested a first loading of 0.4 g/kg of watermelon puree followed by 0.2 g/kg every 15 min of exercise.	20 (all healthy men)	HPLC-UV	Plasma (four collections, pre, post, 1 h post exercice)	Citrulline	[52]
Watermelon juice (3 and 6 cups/day for 3 weeks)	Human controlled crossover study; three intervention periods preceded by 2–4 weeks’ washout period; interventions: (1) control, (2) three cups of watermelon juice a day; (3) six cups of watermelon juice a day.	23 (12 men, 11 women)	HPLC-UV	Plasma (fasting state, baseline, 1 week and 3 weeks of intervention)	Citrulline	[53]
Avocado Assessed by 24 h-dietary recall	Cross-sectional study	301 (129 women, 172men)	GC-MS	Urine (24 h collection)	Perseitol Mannoheptulose	[54]
Avocado (75–200 g of fresh fruit)	Human intervention Study	3 (healthy subjects)	HPLC-QqQ-MS/MS	Urine (prior to consumption and up to 16 h)	Perseitol Mannoheptulose	[55]

*DAD* diode-array-detection, *ECD* electrochemical detection, *ESI* electron spray ionization, *GC* gas-chromatography, *GCxGC* two dimensional-gas chromatography, *HILIC* hydrophilic interaction liquid chromatography, *HPLC* high-performance liquid chromatography, *MS* mass spectrometry, *QqQ* triple quadrupole mass spectrometer, *QTOF* quadrupole-time of flight-mass spectrometer, *QTRAP* quadrupole ion trap, *UHPLC* ultra-performance liquid chromatography, *UV* ultraviolet detection, *6-OH-MTβC-sulfate*, 6-hydroxy-1-methyl-1,2,3,4-tetrahydro-β-carboline sulfate

^a^Food intake reported as median (IQR)

^b^Food intake reported as mean (range of intake)

**Table 2:** Overview of the validation criteria for candidate intake biomarkers for banana and tropical fruits^a^

Food item	Metabolites	Biofluid	Q1	Q2	Q3a	Q3b	Q4	Q5	Q6	Q7	Q8
Banana	5-Hydroxyindole acetic acid	Plasma	Y	U	Y	U	U	U	Y	Y	U
Banana	5-Hydroxyindole acetic acid	Urine	Y	U	Y	U	U	U	Y	Y	U
Banana	3-Methoxytyramine sulfate	Plasma	Y	U	U	U	Y	U	U	U	U
Banana	Dopamine sulfate	Plasma	Y	U	Y	U	Y	U	Y	Y	U
Banana	Dopamine sulfate	Urine	Y	Y*	Y	U	U	U	Y	Y	U
Banana	Methoxyeugenol glucuronide	Urine	Y	Y*	Y	U	U	U	U	U	U
Banana	Salsolinol sulfate	Urine	Y	Y*	Y	U	U	U	U	Y	U
Banana	6-OH-MTβC sulfate	Urine	Y	Y*	U	U	U	U	U	U	U
Banana	Methoxyeugenol glucuronide + Dopamine sulfate	Urine	Y	Y*	Y	U	Y	Y	U	U	U
Banana	Methoxyeugenol-glucuronide + Dopamine sulfate + Salsolinol sulfate	Urine	Y	Y*	Y	U	Y	Y	U	U	U
Banana	Methoxyeugenol glucuronide + Dopamine sulfate + 6-OH-MTβC sulfate + 2-isopropylmalic acid	Urine	Y	Y*	Y	U	Y	Y	U	U	U
Banana	Methoxyeugenol glucuronide + Dopamine sulfate + Salsolinol sulfate + Xanthurenic acid + 6-OH-MTβC sulfate	Urine	Y	Y*	Y	U	Y	Y	U	U	U
Avocado	Perseitol	Urine	Y	U	Y*	U	U	U	U	U	U
Avocado	Mannoheptulose	Urine	Y	U	Y*	U	U	U	U	U	U
Watermelon	Citrulline	Plasma	Y	U	U	U	U	U	U	U	U
Watermelon	Citrulline	Urine	Y	U	U	U	U	U	U	U	U

^a^Questions related to each of the validation criteria: Q1. Is the marker compound plausible as a specific BFI for the food or food group (chemical/biological plausibility)?, Q2. Is there a dose-response relationship at relevant intake levels of the targeted food (quantitative aspect)?, Q3. Is the biomarkers kinetics described adequately to make a wise choice of sample type, frequency and time window (time-response) [a single-meal time-response relationship, b: repeated intakes have]?, Q4. Has the marker been shown to be robust after intake of complex meals reflecting dietary habits of the targeted population (robustness)?, Q5. Has the marker been shown to compare well with other markers or questionnaire data for the same food/food group (reliability)?, Q6. Is the marker chemically and biologically stable during bio specimen collection and storage, making measurements reliable and feasible (stability)?, Q7. Are analytical variability (CV %), accuracy, sensitivity, and specificity known as adequate for at least one reported analytical method (analytical performance)?, Q8. Has the analysis been successfully reproduced in another laboratory (reproducibility)?

*Y* yes the criterion is fulfilled, Y* the criterion is partially fulfilled but requires further investigation, *N* no the principle has not been fulfilled after investigation, *U* unknown; further data is required to determine the validation of the criterion, *6-OH-MTβC sulfate* 6-hydroxy-1-methyl-1,2,3,4-tetrahydro-β-carboline sulfate

### Banana biomarkers

Banana, which is the second most-produced fruit in the world with 113 million tons and is among the three most consumed fruits in Europe [56, 57], certainly deserves specific attention for identifying an intake biomarker. The metabolite 5-hydroxyindole acetic acid (HIAA) was among the most studied compounds in association with banana intake. Of the 23 publications reviewed for this section, 10 reported the impact of banana intake on HIAA in urine (Table 1). HIAA originates from the catabolism of serotonin by the monoamine-oxidase (MAO, EC 1.4.3.4) and aldehyde dehydrogenase (ALDH, EC 1.2.1.3) enzymes. Besides its endogenous origin, serotonin can be provided in substantial amounts by several foods. Banana has a high serotonin content (15 ± 2.4 μg/g FW) along with nuts (87–398 μg/g), pineapple (17 ± 5.1 μg/g FW), plantain (30 ± 7.5 μg/g FW), kiwi fruit (5.8 ± 0.9 μg/g FW), plums (4.7 ± 0.8 μg/g FW), and tomato (3.2 ± 0.6 μg/g FW) [32]. The urinary excretion of HIAA increased by ~ 2–26-fold after banana intakes ranging from 50 to 1000 g [32–40, 49], and was comparable to that induced by an oral dose of 10 mg of serotonin [33]. Along with its sulfated conjugate, HIAA was identified as a highly discriminant compound (VIP > 2; *R*^2^ = 0.89, *Q*^2^ = 0.732) in a randomized, controlled crossover meal study using untargeted metabolomics to discover BFIs for banana [49]. The important contribution of banana intake to HIAA excretion level was also fortuitously observed in a large study aiming at diagnosing neuroblastoma in Japanese 18-month children. Neuroblastoma is a catecholamine-producing tumor, and an elevated excretion of the dopamine metabolite homovanillic acid was used as a diagnosis biomarker. Among the 103 cases identified with elevated homovanillic acid, 50 were false-positive cases caused by regular intake of banana as weaning food, which was associated with elevated excretion levels of both homovanillic acid and HIAA [37].

While serotonin is not specific to banana, its consumption produces a higher elevation of HIAA in urine compared to other serotonin-rich foods, including tomato, pineapple, and kiwi [38, 39]. However, nuts intake constitutes a probable confounder for the use of HIAA as BFI for banana. Feldman et al. reported a higher excretion of HIAA after the ingestion of 100 g of walnuts (7–59 mg/24 h) compared to 100 g of banana (4.8–15 mg/24 h) [32]. Recent studies using untargeted metabolomics proposed HIAA in combination with other metabolites as a candidate biomarker for nuts [58, 59]. Another potential confounder could be fresh tomato, due to its high level of consumption in many populations, but not its processed forms (juices, canned tomato or ketchup) as their serotonin content is much lower (< 0.2 μg/g FW) than in fresh tomato (6.4 μg/g FW) [60]. The robustness of urinary HIAA is thus not fully established. It was not among the metabolites discriminating low and high consumers of banana from non-consumers in a cross-sectional study with 78 subjects [49]. More studies with non-controlled dietary background and examination of the confounding effect of nuts and tomato are needed. While no data are available regarding dose-response, the time-response is well documented for HIAA, with a urinary excretion that peaks 2–4 h after banana intake and returns to baseline levels 8–12 h after consumption [33, 35, 36, 38, 49].

In addition to urine, plasma and serum concentrations of HIAA also increase after banana intake, as reported in four studies [41, 42, 47, 48]. Tohmola et al. measured serum HIAA in response to 35 different foods and showed that walnuts (102 g) produced the highest elevation of HIAA (1.8 μmol/L), followed by banana (~ 0.6 μmol/L for 300 g ingested), pineapple (0.46 μmol/L for 330 g) and tomato (0.39 μmol/L for 362 g) [41]. Plasma concentrations of HIAA peaked 2 h after the intake of all foods and returned to baseline levels 6 h after the intake of tomato and pineapple and 24 h after the ingestion of banana and walnuts. The calculated half-life of HIAA in the circulation was of 1.3 h [41].

Wang et al. conducted a cross-sectional analysis that correlated consumption, using FFQs, of 91 food items or food groups to the non-fasting serum metabolome of 1369 post-menopausal women (Table 1). Herein, HIAA was significantly correlated to banana consumption (|*r*| = 0.21, *P* < 0.0001), although dopamine metabolites showed a better ability to predict banana intake in the same study [48].

Regarding the analytical aspects of the validation scheme for BFIs, quantification of HIAA by HPLC-MS in urine and serum samples is well established [61, 62]. Acidification of urine to a pH between 2 and 5 is recommended for HIAA conservation [63, 64]. At pH 4, HIAA was shown to be stable in urine for up to 30 months at − 20 °C, for 3 months at 4 °C, and for 3 weeks at room temperature [63]. The validation criteria fulfilled so far for HIAA as a biomarker of banana intake are presented in Table 2.

Besides HIAA, the response of dopamine metabolites to banana intake has been widely studied, mostly in plasma and serum [42–44, 46–48], with one publication on urine [49] (Table 1). Banana has a high dopamine content (73 ± 24 μg/g) [38] compared to other dietary sources such as avocado (4 μg/g), orange, cocoa powder, and tomato (< 1 μg/g) [65]. In humans, dopamine is degraded into different metabolites by several pathways [66]. Dopamine-sulfate is the main circulating form originating from both dietary and endogenous dopamine [67, 68]. 3-Methoxytyramine results from the metabolism of dopamine through its methylation by the enzyme catechol-O-methyl transferase (EC 2.1.1.6) [66]; 3-methoxytyramine is further conjugated to its sulfate by the enterocytes or the liver [69].

Dopamine derivatives have been reported as highly distinctive metabolites for banana intake on various occasions. In three intervention studies, banana intake was studied for its effect on the physical endurance of male cyclists, and an untargeted metabolomic analysis of serum samples was included. Herein, dopamine, dopamine-3-O-sulfate, dopamine-4-sulfate, and 3-methoxytyramine-sulfate were detected in plasma as highly discriminant metabolites for the banana intervention [42, 44, 47]. Other authors examined plasma dopamine responses using targeted methods. In a small pilot study, the addition of one banana to a complex meal significantly increased dopamine levels in plasma 1 h after intake from 5.8 ng/mL at baseline to 92.5 ng/mL [43]. A case report about an anorexic adolescent who consumed > 20 bananas/day for 26 months mentioned a 20-fold increase over the normal range of plasma dopamine, which corrected towards normality when the patient resumed the restrictive food ingestion [46]. The latter shows that the intake of banana can increase plasma dopamine to extremely high levels over an extended period of time.

Furthermore, the study by Wang et al. revealed dopamine-3-O-sulfate (*r* = 0.33, *P* < 0.0001; AUC = 0.76), dopamine-4-sulfate (*r* = 0.34, *P* < 0.0001; AUC = 0.74), and 3-methoxytyramine sulfate (*r* = 0.22, *P* < 0.0001; AUC = 0.70) as serum metabolites significantly correlated to banana intake with a good ability to distinguish high and low consumers of banana [48]. These observations support the interest of these three metabolites as candidate BFIs for banana intake in plasma as well as in urine. Their level of validation is discussed below and summarized in Table 2.

In urine samples from a randomized controlled, cross-over meal study, dopamine sulfate was observed as a highly discriminant metabolite (VIP > 2; *R*^2^ = 0.89, *Q*^2^ = 0.732) for banana consumption, and seven-fold higher intensity (*P* < 0.001) after the intake of the fruit than control [49]. Additionally, it was observed in higher intensity in the urine of high consumers of banana compared to non-consumers (3-fold higher; *P* = 6.7 × 10^−3^) in a cross-sectional study on a German population, thus supporting its robustness [49]. Dopamine sulfate was a key component of the combinations of metabolites which performed best to predict banana intake, while its performance as a unique biomarker was lower [49].

Regarding the validation criteria, no clear information is available on the dose-response after banana intake for dopamine metabolites in plasma or urine. The time-response in plasma showed an elevation 1–2 h after the intake of banana, persisting up to 8 h after consumption [43, 70] (Table 2). Urinary excretion of dopamine metabolites peaked 2–4 h after the intake and continued up to 12 h [70]. As for the chemical aspects of the validation, mild acidification of urine samples may be used to avoid catecholamine-oxidation and deconjugation [71]. Sample storage at − 80 °C should be preferred for catecholamine preservation [72]. Liquid chromatographic methods with different detectors, including mass spectrometry, fluorometric, and electrochemical detection, exist for the determination of dopamine, 3-methoxytyramine and its conjugates in plasma and urine [67, 73, 74]. However, conjugated standards of dopamine or 3-methoxytyramine and other compounds are not commercially available for quantification, which may complicate their use as BFIs.

Another metabolite associated with banana intake is salsolinol. This compound is synthesized in banana by the condensation of dopamine and acetaldehyde (Pictet-Spengler reaction) [75]. It is also an endogenous metabolite [76]. Salsolinol concentration in banana pulp is of 6.3 × 10^−2^ μg/g and it is present at lower concentration in other sources including beer (0.5 × 10^−2^ to 1.3 × 10^−2^ μg/g) and French wine (3.3 × 10^−3^ to 4.9 × 10^−3^ μg/g ) [77–79]. Salsolinol is also highly present in cocoa powder (25 μg/g) and chocolate (19 μg/g) [80]. There are two existing isomers of salsolinol, the (R) isomer that is proposed to be mainly of endogenous origin and the (S) enantiomer that may be more susceptible to diet. Strolin-Benedetti et al. reported that in 24 h urine samples of 6 subjects before consumption of banana, the concentration of (S)-salsolinol was close to zero and increased after the intake of dried banana. In contrast, the (R) isomer increased after the intake of the fruit but was already present prior consumption [45]. Lee et al. studied the influence of banana intake on blood concentration of salsolinol in healthy subjects. They showed that after the intake of one banana fruit, the concentration of salsolinol increased from 0.16 ± 0.12 to 5.8 ± 7.6 ng/mL and from 0.23 ± 0.16 to 6.6 ± 8.7 ng/mL, for the (S)- and (R)-forms, respectively, 1.5 h post-consumption [43]. This shows that the consumption of banana causes a detectable increase of salsolinol in plasma. The kinetics of this compound was studied only in rats, after gavage with 10 μg of salsolinol and 3 g of banana. In both interventions, salsolinol increased in plasma 1 h after intake and cleared 14 h post consumption [43].

Salsolinol sulfate was observed as the most distinctive urinary metabolite with a 46-fold higher intensity after a single dose of banana compared to a control meal in 12 healthy subjects (VIP > 2; *R*^2^ = 0.89, *Q*^2^ = 0.732; *P* < 0.001) [49]. In the KarMeN cross-sectional study, this metabolite was also detected in higher intensity in the urine samples of high-consumers compared to non-consumers (10-fold higher in high consumers, *P* = 2 × 10^−2^). However, further analysis showed that salsolinol sulfate alone was not specific enough to predict consumers and non-consumers of banana in this population (misclassification rate > 30%). The robustness of plasma and urine salsolinol as a biomarker of banana intake is possibly challenged by the intake of chocolate, and/or alcohol as their consumption was shown to markedly elevate the levels of salsolinol in biofluids [81]. Intervention studies comparing the response of salsolinol or its conjugates to the intake of different salsolinol-containing foods are needed to further clarify the potential use of salsolinol as a candidate BFI for banana, possibly as a component of a multi-marker.

Among the assessed studies, the one by Vázquez-Manjarrez et al. was specially designed for the discovery and validation of banana biomarkers. Herein, urine samples from two different study designs were analyzed, a controlled, cross-over meal study with 240 g of banana (*n* = 12) and a cross-sectional study (KarMeN) (*n* = 78) with high (126–378 g of banana), low (47.3–94.5 g of banana), and non-consumers of banana using an untargeted multi-platform metabolomics approach [49]. Thirty-three banana-derived metabolites were identified in urine in the meal study, including HIAA, dopamine, and salsolinol metabolites, which have been discussed above, and other highly distinctive metabolites such as methoxyeugenol glucuronide (VIP > 2, *R*^2^ = 0.89, *Q*^2^ = 0.732; 26-fold higher in banana than control, *P* = 1.3 × 10^−10^), 2-isopropylmalic acid (VIP > 2, *R*^2^ = 0.89, *Q*^2^ = 0.732; 9-fold higher in banana than control *P* = 1.4 × 10^−7^), N-acetyldopamine sulfate (VIP > 2, *R*^2^ = 0.89, *Q*^2^ = 0.732; 4-fold higher in banana than control *P* = 1.1 × 10^−6^), 6-hydroxy-1-methyl-1,2,3,4-tetrahydro-β-carboline sulfate (6-OH-MTβC sulfate) (VIP > 2, *R*^2^ = 0.89, *Q*^2^ = 0.732; 4-fold higher in banana than control, *P* = 2.3 × 10^−2^). The cross-sectional KarMeN study showed that methoxyeugenol glucuronide (*P* = 4.70 × 10^−5^), dopamine sulfate (*P* = 6.7 × 10^−3^), salsolinol sulfate (*P* = 2 × 10^−2^), 6-OH-MTβC sulfate (*P* = 3.6 × 10^−2^), and xanthurenic acid (*P* = 6.7 × 10^−3^) had significant higher intensities, ranging from 6- to 25-fold change, in the urine of high-consumers of banana compared to non-consumers, while only methoxyeugenol glucuronide was significantly higher (15-fold, *P* = 5.20 × 10^−5^) in low-consumers compared to non-consumers [49]. None of the putative biomarkers was robust enough when used alone, but the authors demonstrated that dopamine sulfate and methoxyeugenol glucuronide was the best parsimonious combination to detect the intake of banana in high consumers (AUC = 0.92, ER = 0.11) and low consumers (AUC = 0.87, ER = 0.19) [49]. The origin of methoxyeugenol glucuronide was attributed to the metabolism of the characteristic banana aroma methoxyeugenol and elemicin [82]. Methoxyeugenol and elemicin were also reported in nutmeg and star anise [82, 83], respectively, which represent marginal confounders. Regarding dose-response, the intensity of the combined BFIs showed a linear trend with the amount of banana consumed in the intervention study and that reported in 24 h recalls by the high and low consumers in the cross-sectional study. However, quantitative studies are required to fully validate the proposed combined BFI in this critical aspect (Table 2). The metabolite 6-OH-MTβC sulfate was also frequently present in the best combinations of biomarkers reflecting high banana intake in the KarMeN study. The 6-OH-MTβC derives from the condensation of serotonin with acetaldehyde and is present in banana in higher concentrations (1.87 mg/kg) than in other fruits, including tomato (0.71 mg/kg), kiwi (0.31 mg/kg), and pineapple (0.62 mg/kg) [84]. Dark chocolate contains higher levels of 6-OH-MTβC (2.64 mg/kg) [85] than banana and thus would constitute a potential confounder.

Melatonin metabolites have been associated with banana consumption in other selected publications. While melatonin is produced endogenously in the pineal gland, the intake of melatonin-rich foods such as banana, pineapple, cherries, and orange substantially elevate the concentration of melatonin and 6-sulfatoxymelatonin, its main metabolite, in urine and serum [86, 87]. Other dietary sources of melatonin are walnuts, mango, papaya, grapes, wine, and olive oil. The use of melatonin and its main metabolite as a candidate BFI for banana or any other fruit is also hindered by its fluctuation during the daytime [88] and by the fact that melatonin is sold over-the-counter in different countries as a supplement to adjust sleeping disorders.

Many other metabolites have been identified in biofluids after the intake of banana but have less relevance as candidate BFIs (Table S2). 3,4-Dihydroxyphenyl-acetic acid, homovanillic acid, 3-hydroxytyrosol, and 5,6-dihydroxyindole were observed as part of the “dopamine signature” of banana [36, 38, 49]. However, 3,4-dihydroxyphenyl acetic acid and homovanillic acid originate from the microbial metabolism of polyphenols, which are widely distributed in plant-based foods [89, 90]. 3-Hydroxytyrosol in urine may better reflect olive-oil consumption [59]. Plasma 2,3-dihydroxy-isovalerate, 4-guanidinobutanoate, ferulic acid 4-sulfate, and 4-acetylphenol sulfate were associated with banana intake in controlled intervention studies with banana [42, 47]. However, 2,3-dihydroxy-isovalerate is an intermediate in the biosynthesis of valine, leucine, and isoleucine while 4-guanidinobutanoate participates in the metabolic pathway of arginine. Ferulic-acid-4 sulfate is a metabolite of ferulic acid and caffeic acid, which are widely distributed in fruits, cereals, and coffee [28, 30]. 4-Acetylphenol has been detected in different medicinal herbs and is a flavoring and aroma ingredient in roasted coffee, beer, mango, and cranberry [21]. Interestingly 2-isopropylmalic acid, eugenol sulfate, and fructose were observed by different groups after banana intake [42, 47, 49]. Fructose is a common carbohydrate present in a wide range of foods, which excludes its use as a BFI of banana. 2-Isopropylmalic acid has been identified as a key compound for organoleptic characteristics of melon [91] and is present in other vegetables and fruits including lettuce and tomato juice [92, 93]. The fact that this metabolite was recovered in biofluids following consumption of different vegetal sources including pear, banana, and peas [42, 47, 49, 94] suggests its possible utility as putative BFI for the general intake of this food group. Eugenol sulfate results from the phase II metabolism of the aroma compound eugenol which is present in banana and other highly consumed fruits such as apple, apricot, and cherry as well as spices including black pepper and clove [21]. The microbiota metabolites of tryptophan, indolepropionate, and indoleacetate have been associated with the intake of banana [47, 95] but also with the consumption of other fruits including apple and pear [95]. Xanthurenic acid, which was found to be increased in urine after banana intake [49], originates from the kynurenine pathway of tryptophan degradation [96]. Whereas banana has a higher content (10 mg/100 g) of tryptophan than other fruits (e.g., apple 3 mg/100 g) [97], other dietary sources very rich in tryptophan, e.g., cheese and fermented products would act as strong confounders [98].

Late urinary excretion of al N-methyl-2-pyridione-5-carboxylic acid, likely a microbial metabolite of trigonelline, was observed by Vázquez-Manjarrez et al. [49], but the presence of trigonelline in coffee excludes its use as BFI for banana. Metabolites from carboxylic acids, saccharides, and amino acids were also reported in the urine. Among them, sinapic acid-sulfate, hydrocinnamic acid sulfate, vanillic acid, mevalonic acid, azelaic acid, 1,5 anhydrosorbitol, fructose, glyceraldehyde, and 2-ethyl-3-hydroxyl propionic acid were found to be increased in urine samples in studies within a controlled setting but not under free-living conditions [49].

In conclusion, we have identified several candidate BFIs for banana intake including metabolites of catecholamines, indolamines, and specific aroma compounds (Fig. 2). While these metabolites are candidate BFIs for banana, they are challenged when considered individually by their endogenous presence in the human body or in other foods. However, the combined use of several candidate BFIs, namely dopamine sulfate, salsolinol sulfate, 6-OH-MTβC sulfate, and methoxyeugenol glucuronide in urine and HIAA along with 3-methoxytyramine sulfate in plasma, offers a more robust alternative to determine exposure to banana in free-living conditions. These candidate biomarkers can reflect banana intake qualitatively (absence/presence of consumption) but dose-response studies must be conducted to push them forward as quantitative BFIs.

### Papaya and watermelon biomarkers

Much less information has been found on candidate BFIs for tropical fruits other than banana. Some carotenoids have been proposed as biomarkers of tropical fruit intake. In particular, lycopene, β-carotene and β-cryptoxanthin have been associated with papaya and watermelon intake, while lutein and β-carotene have been associated with intake of papaya and mango [99–101]. Tropical fruits have been studied as sources of pro-vitamin A carotenoids for many developing countries with a high occurrence of vitamin A deficiency, and the literature reflects this interest. Carotenes are present in too many foods to be envisaged as biomarkers for specific tropical fruits. β-Cryptoxanthin was shown to increase in plasma after acute intake of papaya [102] and has also been correlated with habitual intake of tropical fruits in a study with 159 Costa-Rican adolescents [101]. Papaya was the best food predictor of plasma β-cryptoxanthin in this study. Yet, although papaya is indeed one of the richest sources of β-cryptoxanthin, this compound can also originate from other orange- or red-colored foods such as orange, mango, apricot, sweet peppers, and pumpkin, as well as from spices and herbs including paprika, dill, and basil [30, 103]. In the Nurse’s Health study conducted in a US population with low consumption of papaya, orange juice was actually reported to be the major determinant of plasma β-cryptoxanthin [104].

Watermelon, and to a lesser extent papaya, contains a substantial amount of lycopene in a highly bioavailable form [102]. Watermelon has a lycopene content ranging from 4.8 to 13.5 mg/100 g fresh weight representing a content equivalent to that measured in most tomato cultivars [103]. However, processed forms of tomato such as tomato sauce are even more concentrated. Considering the higher consumption of tomato products compared to watermelon in most populations, blood concentration of lycopene is more likely to reflect the intake of tomato and derived products than that of watermelon.

Although the common dietary carotenoids lack specificity for tropical fruit intake, it cannot be ruled out that less studied carotenoids among the dozens identified in tropical fruits, for example, cryptoxanthin-5,8-epoxide or sapotexanthin may arise as more specific candidate biomarkers in future metabolomic studies [105].

Aside from carotenoids, the literature search revealed citrulline as a possible biomarker of watermelon intake. Interestingly, the name of this amino acid was derived from the latin name of watermelon, *Citrullus vulgaris*, from which it was first isolated. A case study reported a ten times elevation of citrulline plasma concentration in a young girl who was consuming large quantities of watermelon, and this was confirmed in a dietary intervention on six healthy adult volunteers [50]. Plasma citrulline peaked 1 h after watermelon intake (593 μM), was still elevated at 8 h and returned to the normal range (20–40 μM) at 24 h. Other authors have observed L-citrulline responses in plasma following watermelon intake. A 16-day supplementation with 300 mL/day of a watermelon concentrated juice providing 3.4 g of citrulline/day resulted in 3.5–4.6 fold higher plasma concentrations of citrulline compared to control or apple juice interventions [51]. A transitory increase in plasma L-citrulline occurred for 20 subjects who consumed 0.2 g/kg of watermelon puree every 15 min during a physical challenge, with a return to baseline levels 3–5 h after the end of the supplementation [52]. Collins et al. did not observe any significant change in L-citrulline plasma concentration following the ingestion of watermelon juice, up to 6 cups/day for 3 weeks [53]. However, measurements were made on 12 h fasting plasma samples, which concurs with the short-term responses observed by other authors. Citrulline also has an endogenous origin, synthetized from glutamine or arginine in the enterocytes [106]. It is a key intermediate of the urea cycle in the liver and is readily converted to arginine and then nitric oxide in the kidney and vascular endothelium. Citrulline plasma concentration reflects the equilibrium between its synthesis in the intestine and its conversion to arginine in the kidney. Low or high citrulline status has been proposed as a biomarker of intestinal dysfunction in various intestinal pathologies as well as markers of acute kidney failure and of some inborn errors of the urea cycle [106]. It is worth noting that after watermelon intake plasma arginine was also modestly elevated, whereas venous ammonia, plasma glutamine, and other amino acids remained in the normal range, which does not correspond to the metabolic patterns classically observed with urea cycle, renal or intestinal disorders.

In conclusion, the concentration of citrulline in plasma and possibly in urine could be further explored as candidate biomarker for assessing watermelon intake in healthy individuals (Fig. 2). Regarding papaya, there are no putative BFIs reported in the literature yet. To further discover candidate BFIs for both fruits, controlled intervention trials with different doses of the fruits and applying an untargeted metabolomics approach may be performed.

### Avocado biomarkers

Avocado (*Persea Americana*) is unique in its low content in starch and sugars and high content in monounsaturated fatty acids 71% [107]. Using a semi-targeted GCxGC-MS sugar profiling method, Mack et al. analyzed 24 h urine samples of 301 volunteers of the KarMeN cross-sectional study to search associations between excreted sugar metabolites and the dietary information obtained with 24-dietary recalls [54]. From the Spearman rank correlation analyses with the entire population, two seven-carbon carbohydrates, perseitol (*ρ =* 0.33, *p* values < 0.0001) and mannoheptulose (*ρ =* 0.2702, *p* values < 0.0001) were significantly associated with avocado intake. Further correlation analyses including only avocado consumers (*n* = 9) and non-consumers (*n* = 18) confirmed the strong association of perseitol *ρ* = 0.871 and mannoheptulose *ρ* = 0.778 with avocado consumption [54]*.*

The authors analyzed perseitol and mannoheptulose in 75 foodstuffs to determine their plausibility and specificity as candidate BFIs for avocado. Perseitol was only observed in avocado while mannoheptulose was more concentrated in avocado than in the other fruits and vegetables where it was detected (carrot, blueberry, fig, and tomato) [54].

Wamelink et al. have developed a HPLC-QqQ-MS/MS method of analysis for the seven-carbon carbohydrates and validated it with a small human intervention on 3 subjects consuming avocado (75–200 g) [55]. Urine excretion of perseitol and mannoheptulose but not of other seven-carbon carbohydrates were elevated between 3 and 12 h after avocado intake. A high interindividual variation was observed that may be due to a different content of C7 carbohydrates in avocados. Of note, in subjects with transaldolase deficiency, a recently discovered pentose phosphate pathway alteration, a subtle elevation of mannoheptulose and perseitol in urine can be observed along with other C7 carbohydrates and polyols [55]. This will deserve further attention to determine whether intrinsic factors can affect the excretion of the candidate biomarkers and jeopardize their use.

In conclusion, the limited data available suggest that perseitol and mannoheptulose may be considered as short-term candidate BFIs for avocado. They have been validated for plausibility, and partially for robustness and time-response (Table 2, Fig. 2). However, human studies with larger sample sizes are needed to further validate these candidate BFIs and assess dose-response, reliability, and performance of their quantification method(s).

### Date biomarkers

Date fruit is a highly nutritious fruit that contains about 70% of carbohydrates, as well as dietary fibers and a wide range of micronutrients and secondary metabolites [108, 109]. The first and only study that aimed to identify metabolic changes occurring in blood after consumption of date fruit was conducted by Mathew et al. [110]. They applied an untargeted metabolomics approach based on LC-MS on blood samples from 21 subjects who underwent three dietary interventions: (1) a control glucose drink, (2) ten date fruits of the *Deglet Nour* variety, and (3) ten date fruits of the *Khlas* variety. A total of 28 molecules were found to significantly increase (Bonferroni corrected *p* value < 0.05) over 2 h after the date fruit challenges, among which 20 were identified. Several phenolic acid metabolites were found to be discriminant, including ferulic acid 4-sulfate, caffeic acid-sulfate, 4-vinylguaiacol sulfate, and vanillic alcohol sulfate. This was consistent with the presence of 3,4,5-trimethoxycinnamate, 3,4-dimethoxycinammic acid, 4-hydroxycinnamate, dihydroferulic acid, ferulic acid, and isoferulic acid in the date fruit. However, those phenolic metabolites can derive from many fruits and vegetables and are therefore of little interest as candidate biomarkers for date fruit. In the same study, 2-isopropylmalic acid was observed as a distinctive metabolite for recent date intake. As already mentioned, this compound is also recovered in biofluids after intake of peas or different fruits including banana, tomato, and pear [42, 49, 93, 94]. Some carboxyethylated amino acids were detected in plasma after the intake of both varieties of dates, including 1-carboxyethylisoleucine, 1-carboxyethylleucine, 1-carboxyethyltyrosine, and 1-carboxyethylvaline. These compounds, considered to be advanced glycation products, are formed by non-enzymatic reactions between reducing sugars and proteins [111, 112]. They are most likely formed in the dates during drying, but they might also be formed endogenously after intake of dates as a consequence of their high content of fructose [112].

Based the scarce information available on human metabolites observed after intake of date fruit, it is not yet possible to identify a candidate BFI. Some more specific caffeoylshikimic acids, e.g., dactylifric acid (3-O-caffeoylshikimic acid), neodactylifric acid (5-O-caffeoylshikimic acid), and isodactylifric acid (4- O-caffeoylshikimic acid) resulting from enzymatic browning of date fruit [29, 113] and xanthoxylin [114] may be of interest as putative biomarkers but their metabolic fate in humans remain to be elucidated.

### Pomegranate biomarkers

Pomegranate is mainly consumed fresh or in the form of juice. It is characterized by its content of rare ellagitannins, including punicalin and punicalagin A and B. Thus, it comes as no surprise that most of the retrieved literature focused on urolithins. Urolithins are dibenzo-[*b,d*] pyran-6-one derivatives produced from ellagic acid and ellagitannins by the gut microbiota [115, 116] and to which various beneficial effects on human health have been attributed [117, 118].

After consumption of pomegranate, urolithins appear in plasma 6–8 h after exposure and persist in plasma and urine up to 48–72 h [116, 119–125] (Table S2). It is likely that the primary precursor of urolithins from pomegranate is punicalagin, which constitutes the major ellagitannin present in this fruit.

Two main factors limit the usefulness of urolithins as BFIs for pomegranate. Firstly, their precursors, namely ellagitannins and ellagic acid, are distributed in other highly consumed foods, including berries and walnuts. The estimated dietary contribution of total ellagitannins as assessed by 3-day dietary records and chromatographic analysis revealed five primary dietary sources of ellagitannins: strawberries, blackberries, walnuts, pomegranate juice, and preserved foods [123]. Of these foods, berries accounted for almost half (42%) of the estimated intake of ellagitannins, followed by pomegranate (27.6%), walnuts (26.7%), and strawberry jam (4.1%). Tea infusions (*Camelia Sinensis*) also contain ellagitannins and ellagic acid [126]. The high frequency of consumption of tea, berries, and walnuts makes them potential confounders for the use of urolithins as BFIs for pomegranate. The second limiting factor is the high inter-individual variation in the production of urolithins caused by variable gut microbiota functionality [127]. From the analysis of urine samples of subjects involved in different dietary interventions with walnuts, pomegranate, or berries, the group of Tomás-Barberan and others reported the occurrence of three ellagitannin-metabolizing phenotypes: metabotype A, metabotype B, and metabotype 0 [115–117, 124, 127–131]. Metabotype A subjects, representing 25–80% of the tested populations, produce solely urolithin A conjugates. Metabotype B found in 10–50% of the volunteers produce isourolithin A and urolithin B besides urolithin A. Lastly, subjects with metabotype 0, less than 10% of the population, do not produce any of these urolithins.

In conclusion, urolithins are not promising candidate BFIs for pomegranate and additional work has to be done to identify specific BFIs that would apply for all subjects regardless of their metabotype. As no other specific pomegranate component has been highlighted so far, the best approach to identify putative BFIs for pomegranate is to conduct an explorative intervention study with untargeted metabolomic profiling of the collected biofluids and later validate the robustness of the discovered candidates in a population in free-living conditions.

### Mango biomarkers

Mango is a rich source of phytochemicals including diverse polyphenols such as catechins, quercetin, kaempferol, rhamnetin, anthocyanins, gallic acid, ellagic acid, protocatechuic acid, and benzoic acid, which are all widely distributed in other fruits [132, 133]. Gallic acid has been found as the most abundant in mango pulp [134] along with different polymers of the same metabolite in the form of gallotannins. Barnes et al. studied the bioavailability of galloyl derivatives after consumption of mango (Keitt variety). Eleven healthy volunteers underwent a dietary intervention with 400 g of mango daily for 10 days. Plasma and urine samples were collected on the first and last days of intervention. While no response was observed in plasma, seven metabolites of gallic acid and pyrogallol derivatives were detected in urine [134]. The gallic acid metabolites 4-methylgallic acid and 4-O-methylgallic acid-3-O-sulfate accounted for 43–54% of the total administered gallic acid and showed a rapid urinary excretion peaking at 6 h and returning to baseline 8–12 h after the intake of mango [134]. The other five metabolites were pyrogallol conjugates that originate from the microbial decarboxylation of gallic acid, namely isoforms of *O-*methylpyrogallol-*O-*sulfate, pyrogallol-*O-*sulfate, and deoxypyrogallol-*O-*sulfate [135]. These five metabolites had a urinary excretion peaking at 12 h after the consumption of mango. After 10 days with 400 g/day of mango, pyrogallol-*O-*sulfate and deoxypyrogallol-*O-*sulfate were the most prevalent metabolites with a significantly increased urinary excretion (> 60%, *P* < 0.05).

Quirós-Sauceda et al. reported consistent results on pyrogallol metabolites in hydrolyzed plasma and urine samples of 12 male volunteers after the intake of a different variety of mango (Ataulfo) [133]. Five compounds, i.e., gallic acid, chlorogenic acid, protocatechuic acid, ferulic acid, and genistic acid, all unspecific for mango intake, were detected in hydrolyzed plasma 2–4 h after the intake of either 500 g of mango pulp or 721 g of mango juice. In urine, gallic acid, pyrogallol, chlorogenic acid, vanillic acid, p-coumaric acid, ferulic acid, and sinapic acid were detected [133]. Most metabolites showed a rapid urinary excretion while pyrogallol was observed over 8–24 h after the intake of mango in both forms.

None of the aforementioned phenolic metabolites may be considered as candidate BFIs for mango due their low specificity for this fruit. Pyrogallol and its conjugates have been detected in human biofluids following the intake of other fruit products, such as grape juice and berries [136, 137]. Mangiferin, a xanthonoid, has been isolated from the pulp of different mango varieties including the Keitt and Ataulfo used in the studies discussed in this section [132]. While its presence and specificity for mango makes it plausible candidate BFI for mango, no information is available yet on the bioavailability of this compound. Its combination with metabolites of pyrogallol may be further tested as a candidate multi-marker for mango.

### Potential biomarkers for other tropical fruits

In spite of the frequent consumption of kiwi, muskmelon, pineapple, guava, and persimmon, there is a remarkable paucity of information on their metabolites in human studies, and even fewer data are available for the less common tropical fruits acerola, litchi, pitaya (dragon fruit), and coconut.

Abdul et al. examined the metabolic profiling of three varieties of kiwi including the most consumed Hayward cultivar (A*ctinidia deliciosa*), the mini kiwi (*Actindia arguta*), and the less known Bidan (*Actinidia eriantha*) [138]. Only non-specific compounds were detected, including phenylalanine, tyrosine, arginine, citric acid, glutamine-hydroxy-L-proline, 4-aminobutyrate (GABA), glutamate, glutamine, quinic acid, actinic acid, shikimate, mannose, syringic acid, and afzelechin [22, 138].

Muskmelon, or cantaloupe, is a widely consumed fruit in different countries. Besides their content in β-cryptoxanthins, other interesting phytochemicals reported in this fruit are cucurbitin and cucurbitacin E and B [21, 22, 139] (Figure S1). Little is known about the bioavailability of these compounds after consumption of muskmelon, and they have also been reported in other fruits of the cucurbitacae family, such as cucumber or pumpkin [22, 24]

Regarding guava, while no publications were retrieved that report specific metabolites following its intake, some specific terpenes have been described in the fruit. Qin et al. reported the presence of guadial, guajadial, 4,5-diepipsidial, psiguadial, and two novel meroterpenoids, psiguajavadial A and B [140]. Although studies on the bioavailability and pharmacokinetics of terpenes in humans are scarce, evidence indicating their bioavailability after the intake of other fruits exists [141, 142]. Thus, these metabolites might constitute putative biomarkers for the intake of guava (Figure S1). Moreover, the presence of mongolicain-A, an ellagitannin, has been reported solely in guava [22, 29] advocating for a potential role as a putative BFI for this fruit. However, as discussed previously its bioavailability in humans in not documented and it may be metabolized into non-specific compounds like urolithins with a high interindividual variation.

Pineapple belongs to the *Ananas cosmosus* species. Like in other tropical fruits, melatonin is present in pineapple and its consumption has been shown to produce an elevation of 6-sulfatoxymelatonin in urine of healthy adults [143]. However, as discussed in the “Banana biomarkers” section this metabolite is not relevant as a BFI for either pineapple or any other fruit due to its natural abundance in many fruits and in supplements. Ananasic acid, a specific triterpenoid reported in pineapple [21, 22, 144], may be further studied for its plausibility as a putative BFI for pineapple.

Leucodelphinidin-3-glucoside [145], kakidiol, and methyl-phaeophorbide A and B are listed in different food databases for persimmon [21–23] (Figure S1). Information on the bioavailability of these compounds after human consumption of persimmon is not available. Finally, no specific phytochemicals were identified for litchi, pitaya (dragon fruit), coconut, or acerola in our review process.

## Conclusion

In spite of the important presence of tropical fruits in the human diet, there has been very little interest so far for biomarkers of their intake, and except for banana, no studies have been specifically designed for identifying such biomarkers. For banana consumption, dopamine sulfate, salsolinol sulfate, 6-OH-MTβC sulfate, methoxyeugenol-glucuronide, HIAA, and 3-methoxytyramine sulfate are candidate biomarkers. The combination of some of these individual compounds may strengthen their robustness to overcome the exposure to potential confounders, as demonstrated for the combination of methoxyeugenol glucuronide and dopamine sulfate in the urine. However, dose-response studies are needed to validate the latter combination as an accurate quantitative BFI for banana. Citrulline is a candidate BFI for watermelon but its robustness, especially given its endogenous formation in the gut epithelium, has to be assessed in population studies. Perseitol and mannoheptulose are promising candidate BFIs for avocado but again, larger population studies are required to document their robustness and reliability. A few human intervention studies with mango, papaya, pomegranate, and dates have been performed, but no candidate BFIs were identified so far for these fruits. Specific phytochemicals have been described in some tropical fruits including mango, pineapple, guava, and persimmon, but there is virtually no information regarding their bioavailability and interest as putative BFIs.

The way forward for discovering BFIs for tropical fruits is certainly the application of untargeted metabolomics, where no a priori hypothesis of candidate biomarkers is required. Complementary studies with different study design, such as controlled cross-over trials with different doses of the fruits, and cross-sectional studies with comprehensive dietary information will have to be used to document the criteria of the validation scheme for BFIs.

In parallel, data collection and sharing in online resources on the phytochemical composition of tropical fruits and the bioavailability of these phytochemicals should facilitate the identification of specific phytochemical metabolites in metabolomic profiles that may constitute new candidate BFIs.

## Supplementary information

**Additional file 1: Table S1.** Validation criteria for biomarkers of food intake as established by Dragsted et al.

**Additional file 2: Table S2.** Summary of selected candidate, putative and excluded biomarkers of food intake tropical fruits, measured in biofluids^1^.

**Additional file 3: Figure S1.** Specific compounds found in different tropical fruits that may be further explored as putative BFIs in human studies.

## Data Availability

Not applicable.

## Abbreviations

BFIRev: Biomarker of Food Intake Reviews
BFI: Biomarker of food intake
HILIC-QTRAP-MSMS: Hydrophilic interaction liquid chromatography-quadrupole ion trap-mass spectrometry
HPLC: High-performance liquid chromatography
DAD: Diode-array-detection
ECD: Electrochemical detection
ESI-MS: Electron spray ionization-mass spectrometry
UV: Ultraviolet detection
MS: Mass spectrometry
QqQ: Triple quadrupole- mass spectrometry
GC: Gas-chromatography
GCxGC: Two dimensional-gas chromatography
UHPLC: Ultra-performance liquid chromatography
QTOF: Quadrupole-time of flight-mass spectrometry
6-OH-MTβC-sulfate: 6-Hydroxy-1-methyl-1,2,3,4-tetrahydro-β-carboline sulfate
