# Supplementary material for: Biomarkers of intake for tropical fruits
Source: Genes Nutr. 2020 Jun 19;15:11. doi: 10.1186/s12263-020-00670-4 (PMC7304196; doi:10.1186/s12263-020-00670-4)
Supplement: Supplementary file 1 — Additional file 1: Table S1. Validation criteria for biomarkers of food intake as established by Dragsted et al. [file 12263_2020_670_MOESM1_ESM.docx]

# Supplementary Table 1. Validation criteria for biomarkers of food intake as established by Dragsted et al.

| **Validation Criterion** | **Question** | **Possible answers^1^** |
| --- | --- | --- |
| Plausibility | Q1. Is the marker compound plausible as a specific BFI for the food or food group (chemical/biological plausibility)? | Y N U |
| Dose-response | Q2. Is there a dose-response relationship at relevant intake levels of the targeted food (quantitative aspect)? | Y N U |
| Time response (single dose) (multiple dose) | Q3.Is the biomarkers kinetics described adequately to make a wise choice of sample type, frequency and time window (time-response)?  a:The single-meal time-response relationship of the BFI has been described for a defined sample type and time window in a meal study.  b: The kinetics of the BFI after repeated intakes have been described for a defined sample type in a meal study OR accumulation of the BFI in  certain sample types has been observed. | Y N U |
| Robustness | Q4.Has the marker been shown to be robust after intake of complex meals reflecting dietary habits of the targeted population (robustness)? | Y N U |
| Reliability | Q5.Has the marker been shown to compare well with other markers or questionnaire data for the same food/food group (reliability)? | Y N U |
| Stability | Q6.Is the marker chemically and biologically stable during bio specimen collection and storage, making measurements reliable and feasible (stability)? | Y N U |
| Analytical performance | Q7.Are analytical variability (CV %), accuracy, sensitivity, and specificity known as adequate for at least one reported analytical method (analytical performance)? | Y N U |
| Reproducibility | Q8. Has the analysis been successfully reproduced in another laboratory (reproducibility)? | Y N U |

^1^Y, yes the principle is fulfilled; Y*, the principle is partially fulfilled and requires further investigation; N, the principle has not been fulfilled after investigation; U, unknown further data is required to determine the validation of the principle
