# Supplementary material for: Biomarkers of intake for tropical fruits
Source: Genes Nutr. 2020 Jun 19;15:11. doi: 10.1186/s12263-020-00670-4 (PMC7304196; doi:10.1186/s12263-020-00670-4)
Supplement: Supplementary file 2 — Additional file 2: Table S2. Summary of selected candidate, putative and excluded biomarkers of food intake tropical fruits, measured in biofluids1. [file 12263_2020_670_MOESM2_ESM.docx]

# Table S2. Summary of selected candidate, putative and excluded biomarkers of food intake tropical fruits, measured in biofluids^1^.

| **Food Item** | **Compound** | **Phytohub Identifier ^1^** | **HMDB Identifier ^1^** | **Biofluid** | **Candidate/Putative BFI** | **Reasons for inclusion/exclusion *** | **References** |
| --- | --- | --- | --- | --- | --- | --- | --- |
| Avocado | Perseitol | PHUB001956 | HMDB0033750 | Urine | Yes | See text | (1, 2) |
| Avocado | Mannoheptulose | - | HMDB0029935 | Urine | Yes | See text | (1, 2) |
| Banana | 5-hydroxindole acetic acid | PHUB001475 | HMDB0000763 | Urine/ Plasma | Yes | See text | (3-15) |
| Banana | Dopamine-sulfate | PHUB001951  PHUB001952 | HMDB0006275  HMDB0004148 | Urine/ Plasma | Yes | See text | (12-15) |
| Banana | Salsolinol-sulfate | PHUB001950 | - | Urine | Yes | See text | (14) |
| Banana | Methoxyeugenol-glucuronide | PHUB001953 | - | Urine | Yes | See text | (14) |
| Banana | 6-OH-MTβC-sulfate | PHUB001954 | - | Urine | Yes | See text | (14) |
| Banana | 3-methoxytyramine sulfate | PHUB001958 | - | Plasma/Urine | Yes | See text | (13-15) |
| Banana | 5-hydroxytryptophol | - | HMDB01855 | Urine | No | Low response after intake of banana | (5, 16) |
| Banana  Dates | 2-isopropylmalic acid | PHUB001957 | HMDB00402 | Urine/ Plasma | No | Unspecific for banana; lacked robustness in a cohort study; also observed as highly distinctive following the intake of peas. | (13, 14, 17) |
| Banana  Date | 4-acetylphenol sulfate | PHUB001959 | - | Plasma | No | Unspecific | (13, 17) |
| Banana | Xanthurenic acid | - | HMDB0000881 | Urine | No | Metabolite of tryptophan. Not specific for banana intake | (14) |
| Banana | Tyramine O-sulfate | PHUB001960 | HMDB06409 | Plasma | No | Low response after intake, has not been sufficiently studied. Putative BFI for cheese. | (13) |
| Banana | Fructose | - | HMDB00660 | Plasma | No | Unspecific for food or food group | (13) |
| Banana | Eugenol sulfate | - | HMDB05809 | Urine/ Plasma | No | Metabolite of eugenol which is present in several fruits, lacks robustness in cohort study | (13, 14) |
| Banana  Date | Vanillic alcohol sulfate | - | HMDB32012 | Urine/ Plasma | No | Unspecific for banana | (13, 14, 17) |
| Banana  Date | 4-guanidinobutanoate | - | HMDB03464 | Plasma | No | Unspecific for food or food group | (13, 17) |
| Banana  Date | Ferulic acid-4 sulfate | PHUB001171 | HMDB29200 | Plasma | No | Unspecific for food or food group | (12, 13, 17) |
| Banana  Pineapple | Melatonin | PHUB001644 | HMDB01389 | Serum | No | Unspecific for banana; present after the intake of other fruits including cherry and pineapple; it is also sold over the counter as sleep-remedy. | (18) |
| Banana | 6-sulfatoxymelatonin | - | HMDB41815 | Urine | No | Unspecific | (18, 19) |
| Banana | Vanillylmandelic acid | - | HMDB00291 | Urine | No | Low response after intake of banana, lacks robustness | (10) |
| Banana | 3-4 Dihydroxyphenyl acetic acid | PHUB000527 | HMDB01336 | Urine | No | Endogenous dopamine metabolite, also has a microbiota origin from polyphenol catabolism, lacked robustness in the cohort study | (10, 14) |
| Banana | Homovanillic acid | PHUB000617 | HMDB00118 | Urine | No | Unspecific for banana, lacked robustness in cross-sectional studies. | (10, 14, 20) |
| Banana | Indolepropionate | - | HMDB0002302 | Plasma | No | Microbiota metabolite of tryptophan catabolism also associated with apple and pear. Lacks robustness for banana. | (21) |
| Banana | N-Acetyldopamine sulfate | PHUB001974 | - | Urine | No | Endogenous metabolite, while it was observed as highly distinctive in the intervention study it lacked robustness in cohort study | (14) |
| Banana | Kynurenic acid | - | HMDB0000715 | Urine/ Serum | No | Endogenous metabolite, tryptophan metabolite, while it was observed as highly distinctive in intervention studies it lacked robustness in cohort studies, not specific enough for banana or any other fruit | (12, 14) |
| Banana | Fructose derivatives | - | HMDB0000660 | Urine | No | Unspecific for banana, common to many fruits | (14) |
| Banana | N-Methyl-2-pyridone-5-carboxylic acid | - | - | Urine | No | Unspecific for banana, trigonelline metabolite, lacks robustness in cohort study | (14) |
| Banana | Norepinephrine | PHUB000779 | HMDB0000216 | Urine | No | Endogenous metabolite, lacked robustness in a cohort study | (14) |
| Banana | 1,5-Anhydrosorbitol | - | HMDB0002712 | Urine | No | Showed low response following the intake of banana; unspecific | (14) |
| Banana | 2-Ethyl-3-hydroxypropionic acid | - | HMDB0000396 | Urine | No | Showed low response following the intake of banana; unspecific | (14) |
| Banana | Homovanillic acid | PHUB000617 | HMDB0000118 | Urine | No | Dopamine metabolite, endogenous metabolite, lacked robustness in cohort study | (14) |
| Banana | Methoxyeugenol sulfate | PHUB001961 | - | Urine | No | Metabolite of methoxyeugenol insufficient information regarding its robustness | (14) |
| Banana | Azelaic acid | - | HMDB0000784 | Urine | No | Unspecific to banana, lacked robustness in cohort studies, also present in whole grain cereals | (14) |
| Banana | Mevalonic acid | - | HMDB0000227 | Urine | No | Endogenous metabolite, lacked robustness in cohort study, unspecific to banana. | (14) |
| Banana | Sinapic acid sulfate | PHUB001962 | - | Urine | No | Hydrocinnamic acid metabolite, unspecific for banana, lacked robustness in cohort study | (14) |
| Banana | Hydrocinnamic acid sulfate | PHUB001964 | - | Urine | No | Metabolite of hydrocinnamic acid, lacked robustness in cohort study, unspecific for banana | (14) |
| Banana  Date | Hippuric acid | PHUB001174 | HMDB0000714 | Urine/ Serum | No | Common polyphenol metabolite, unspecific for banana, commonly reported in urine following the intake of other fruits and vegetables. | (13, 14, 17) |
| Banana | Glyceraldehyde | - | HMDB0001051 | Urine | No | Unspecific for banana, showed a low response to the intake of banana. | (14) |
| Banana | 3-Hydroxytyrosol | PHUB001321 | HMDB0005784 | Urine | No | Dopamine metabolite, lacked robustness in the cohort study, unspecific for banana, also significantly present following the intake of olives and olive oil | (14) |
| Banana | 5,6-Dihydroxyindole | - | HMDB0004058 | Urine | No | Dopamine metabolite, lacked robustness in the cohort study | (14) |
| Banana | Norepinephrine | PHUB000779 | HMDB0000216 | Urine | No | Lacked robustness in the cohort studies | (3, 14) |
| Banana | 5-bromotryptophan | - | - | Plasma | No | Endogenous metabolite, not specific | (12) |
| Banana | Indoleacetate | - | HMDB0000197 | Plasma | No | Microbiota metabolite from tryptophan, lacks robustness, unspecific to banana | (12) |
| Banana | 3-indoxyl-sulfate | - | HMDB0000682 | Plasma | No | Tryptophan metabolite, lacks robustness, unspecific of banana. | (12) |
| Banana | Argininate | PHUB001965 | - | Plasma | No | Urea cycle metabolite, unspecific for banana | (12) |
| Banana | Trans-4-hydroxyproline | - | HMDB0000725 | Plasma | No | Urea cycle metabolite, unspecific for banana | (12) |
| Banana | 2-oxoarginine | - | HMDB0004225 | Plasma | No | Urea cycle metabolite, unspecific for banana | (12) |
| Banana | Proline | - | HMDB0000162 | Plasma | No | Non-essential amino acid, unspecific for banana | (12) |
| Banana | S-methylmethionine | - | HMDB0038670 | Plasma | No | Unspecific for banana also detected in brassicas and other fruits and vegetables | (12) |
| Banana | Pyridoxate | - | HMDB0000017 | Plasma | No | Metabolite of Vitamin B6, unspecific for banana | (12) |
| Date | Alanine | - | HMDB0000161 | Plasma | No | Amino acid present in several foods, endogenous metabolite. | (17) |
| Date | Creatine | - | HMDB0000064 | Plasma | No | Endogenous metabolite, not specific. | (17) |
| Date | Betaine | - | HMDB0000043 | Plasma | No | Endogenous metabolite, not specific. | (17) |
| Date | Pipecolate | - | HMDB0000070 | Plasma | No | Not specific for food intake, possible a microbiota metabolite from lysine metabolism. | (17) |
| Date | 5-hydroxymethyl-2-furoic acid | PHUB001892 | HMDB0002432 | Plasma | No | Endogenous metabolite from amino acid metabolism, not specific. | (17) |
| Date | 1-carboxyethyl-leucine | - | - | Plasma | No | Advanced glycation product, multiple dietary sources for this metabolite exist, including endogenous metabolism. | (17) |
| Date | 1-carboxyethyl-valine | - | - | Plasma | No | Advanced glycation product, multiple dietary sources for this metabolite exist, including endogenous metabolism | (17) |
| Date | 1-carboxyethyl-tyrosine | - | - | Plasma | No | Advanced glycation product, multiple dietary sources for this metabolite exist, including endogenous metabolism | (17) |
| Date | 1-carboxyethyl-isoleucine | - | - | Plasma | No | Advanced glycation product, multiple dietary sources for this metabolite exist, including endogenous metabolism | (17) |
| Date | 1-carboxyethyl-phenylalanine | - | - | Plasma | No | Advanced glycation product, multiple dietary sources for this metabolite exist, including endogenous metabolism | (17) |
| Date | Erythronate | - | HMDB0000613 | Plasma | No | Endogenous metabolite; lacks specificity. | (17) |
| Date | Sucrose |  | HMDB0000258 | Plasma | No | Common sugar found in multiple dietary sources. | (17) |
| Date | Fructose | - | HMDB0000660 | Plasma | No | Common monosaccharide found in multiple dietary sources, not specific. | (17) |
| Date | Glucose | - | HMDB0000122 | Plasma | No | Common monosaccharide found in multiple dietary sources, not specific. | (17) |
| Date | Succinyl-carnitine | - | HMDB0061717 | Plasma | No | Endogenous metabolite, not specific. | (17) |
| Date | Carnitine | - | HMDB0000062 | Plasma | No | Endogenous metabolite, not specific. | (17) |
| Date | 4-hydroxy-2-oxoglutaric acid | - | HMDB0002070 | Plasma | No | Endogenous metabolite, not specific | (17) |
| Date | 1-oleoyl-GPG (18:1) | - | - | Plasma | No | Unspecific for date, endogenous metabolite. | (17) |
| Date | 3-hydroxy-3-methylglutarate | - | HMDB0000355 | Plasma | No | Endogenous metabolite, not specific | (17) |
| Date | 3-methylcytidine | - | HMDB0240577 | Plasma | No | Endogenous metabolite, not specific | (17) |
| Date | Benzoyl-carnitine | - | - | Plasma | No | Endogenous metabolite, not specific | (17) |
| Date | 2-pyrrolidinone | - | HMDB0002039 | Plasma | No | Endogenous metabolite, not specific | (17) |
| Date | Pyrraline | - | HMDB0033143 | Plasma | No | Maillard reaction metabolite, not specific. | (17) |
| Date | 2-keto-3-deoxy-gluconate | - | HMDB0001353 | Plasma | No | Endogenous metabolite, not specific | (17) |
| Date | N-(2-furoyl)glycine |  | HMDB0000439 | Plasma | No | Endogenous metabolite, not specific | (17) |
| Date | 4-vinylguaiacol sulfate | PHUB001966 | HMDB0127980 | Plasma | No | Unspecific for date also detected in other fruits and in beer. | (17) |
| Date | Caffeic acid sulfate | PHUB001594 | HMDB0041706 | Plasma | No | Common metabolite of polyphenols, not specific for date | (17) |
| Mango | Pyrogallol | PHUB000632 | HMDB0013674 | Urine | No | Microbiota metabolite of gallic acid resulting from decarboxylation, high inter-individual variability, | (22) |
| Mango | 4-O-Methylgallic acid | PHUB001861 | HMDB0013198 | Urine/ Plasma | No | Microbiota metabolite of gallic acid, high inter-individual variability, low specificity for mango. | (23) |
| Mango | 4-Methylgallic acid-3-0-sulfate | PHUB001873 | - | Urine/ Plasma | No | Conjugate of microbiota metabolite of gallic acid, high inter-individual variability, and low specificity for mango. | (23) |
| Mango | Pyrogallol-1-O-sulfate | PHUB001967 | HMDB0060016 | Urine/ Plasma | No | Conjugate of the microbiota metabolite, pyrogallol, high inter-individual variability, and low specificity for mango. | (23) |
| Mango | Pyrogallol-2-O-sulfate | PHUB001416 | HMDB0060018 | Urine/ Plasma | No | Conjugate of the microbiota metabolite, pyrogallol, high inter-individual variability, and low specificity for mango. | (23) |
| Mango | Deoxypyrogallol-O-sulfate | PHUB001968 | - | Urine/ Plasma | No | Conjugate of the microbiota metabolite, pyrogallol, high inter-individual variability, and low specificity for mango. | (23) |
| Mango | Methylpyrogallol-O-sulfate | PHUB001969 | - | Urine/ Plasma | No | Conjugate of a microbiota metabolite of gallic acid, high inter-individual variability, and low specificity for mango. | (23) |
| Mango  Papaya | Lutein | PHUB000361 | HMDB0003233 | Plasma/ Chylomicrons | No | Low specificity for mango or any other fruit. | (24) |
| Pomegranate | Urolithin A | PHUB001391 | HMDB0013695 | Plasma/ Urine/ Faeces | No | Microbiota metabolite of ellagitannins, high inter-individual variability, and low specificity for pomegranate, reported after the intake of e.g. berries and walnuts. | (25-31) |
| Pomegranate | Urolithin B | PHUB001394 | HMDB0013696 | Plasma/ Urine/ Faeces | No | Microbiota metabolite of ellagitannins, high inter-individual variability, low specificity for pomegranate, it has been reported after the intake of e.g. berries and walnuts. | (25-32) |
| Pomegranate | Urolithin C | PHUB001396 | HMDB0029218 | Urine/ Plasma | No | Microbiota metabolite of ellagitannins, high inter-individual variability, and low specificity for pomegranate, it has been reported after the intake of e.g. berries and walnuts. | (29) |
| Pomegranate | Ellagic acid | PHUB000298 | HMDB002899 | Urine/ Plasma | No | Not specific for pomegranate reported after the intake of other highly consumed fruits, such as berries. | (29, 30, 33-35) |
| Pomegranate | Isourolithin A | PHUB001647 | - | Urine | No | Microbiota metabolite of ellagitannins, high inter-individual variability, and low specificity for pomegranate, it has been reported after the intake of e.g. berries and walnuts. | (26) |
| Pomegranate | Urolithin A glucuronide | PHUB001392 | HMDB0029222 | Urine/ Plasma | No | Conjugate of microbiota metabolite of ellagitannins, high inter-individual variability, low specificity for pomegranate, it has been reported after the intake of e.g. berries and walnuts. | (25, 26, 28, 30, 31, 35-37) |
| Pomegranate | Urolithin B glucuronide | PHUB001395 | HMDB0041787 | Urine/ Plasma | No | Conjugate of microbiota metabolite of ellagitannins, high inter-individual variability, low specificity for pomegranate, it has been reported after the intake of e.g. berries and walnuts. | (25, 26, 28, 30, 31, 33-37) |
| Pomegranate | Urolithin A-sulfate | PHUB001970 | - | Urine/ Plasma | No | Conjugate of microbiota metabolite of ellagitannins, high inter-individual variability, low specificity for pomegranate, it has been reported after the intake of e.g. berries and walnuts. | (25) |
| Pomegranate | Urolithin B-sulfate | PHUB001971 | - | Urine/ Plasma | No | Conjugate of microbiota metabolite of ellagitannins, high inter-individual variability, low specificity for pomegranate, it has been reported after the intake of e.g. berries and walnuts. | (25) |
| Pomegranate | Isourolithin A glucuronide | PHUB001648 | - | Urine/ Plasma | No | Conjugate of microbiota metabolite of ellagitannins, high inter-individual variability, low specificity for pomegranate, it has been reported after the intake of e.g. berries and walnuts. | (25, 26, 31, 34, 36) |
| Pomegranate | Isourolithin A sulfate | PHUB001972 | - | Plasma | No | Conjugate of microbiota metabolite of ellagitannins, high inter-individual variability, low specificity for pomegranate, it has been reported after the intake of e.g. berries and walnuts. | (34) |
| Pomegranate | Dimethylellagic acid glucuronide | PHUB001973 | - | Urine | No | Conjugate of microbiota metabolite of ellagic acid and ellagitannins, low specificity for pomegranate | (30, 33, 35) |
| Pomegranate | Hydroxy-urolithin A | - | - | Urine | No | Microbiota metabolite of ellagitannins, high inter-individual variability, low specificity for pomegranate, it has been reported after the intake of e.g. berries and walnuts | (30) |
| Watermelon | Citrulline | PHUB001955 | HMDB0000904 | Urine/ Plasma | Yes | See text | (38-41) |
| Watermelon  Papaya | Lycopene | PHUB000363 | HMDB0003000 | Plasma | No | Low specificity for mango or any other fruit | (42, 43) |
| Watermelon  Papaya | β-Cryptoxanthin | PHUB000356 | HMDB0033844 | Plasma/  Triglyceride-rich lipoproteins | No | Low specificity for mango or any other fruit | (43, 44) |
| Watermelon  Papaya | β-Carotene | PHUB000350 | HMDB0000561 | Plasma/  Chylomicrons | No | Low specificity for mango or any other fruit | (24, 42) |
| Watermelon  Papaya  Mango | α−Carotene | PHUB000349 | HMDB0003993 | Plasma/  Chylomicrons | No | Low specificity for mango or any other fruit | (24) |

^1^ BFI, biomarker of food intake ; HMDB, Human metabolome database <http://www.hmdb.ca/>; Phytohub, <http://phytohub.eu/>. * Excluded markers might still be useful in certain combinations together with more specific markers.

# Supplementary References

1. Mack CI, Weinert CH, Egert B, Ferrario PG, Bub A, Hoffmann I, Watzl B, Daniel H, Kulling SE. The complex human urinary sugar profile: determinants revealed in the cross-sectional KarMeN study. Am. J. Clin. Nutr. 2018;108(3):502-16.

2. Wamelink MM, Smith DE, Jansen EE, Verhoeven NM, Struys EA, Jakobs C. Detection of transaldolase deficiency by quantification of novel seven-carbon chain carbohydrate biomarkers in urine. J. Inherit. Metab. Dis. 2007;30(5):735-42.

3. Crout JR, Sjoerdsma A. The Clinical and laboratory significance of serotonin and cathecolamines in bananas. N. Engl. J. Med. 1959;261(1):23-6.

4. Feldman JM, Lee EM. Serotonin content of foods: effect on urinary excretion of 5-hydroxyindoleacetic acid. Am. J. Clin. Nutr. 1985;42(4):639-43.

5. Helander A, Some M. Dietary serotonin and alcohol combined may provoke adverse physiological symptoms due to 5-hydroxytryptophol. Life Sci. 2000;67(7):799-806.

6. Helander A, Wikstrom T, Lowenmo C, Jacobsson G, Beck O. Urinary excretion of 5-hydroxyindole-3-acetic acid and 5-hydroxytryptophol after oral loading with serotonin. Life Sci. 1992;50(17):1207-13.

7. Odink J, Korthals H, Knijff JH. Simultaneous determination of the major acidic metabolites of catecholamines and serotonin in urine by liquid chromatography with electrochemical detection after a one-step sample clean-up on Sephadex G-10; influence of vanilla and banana ingestion. J. Chromatogr. 1988;424(2):273-83.

8. Numata K, Kusui H, Kawakatsu H, Kizaki Z, Sawada T. Increased urinary HVA levels in neuroblastoma screens related to diet, not tumor. Pediatr. Hematol. Oncol. 1997;14(6):569-76.

9. Tohmola N, Johansson A, Sane T, Renkonen R, Hamalainen E, Itkonen O. Transient elevation of serum 5-HIAA by dietary serotonin and distribution of 5-HIAA in serum protein fractions. Ann. Clin. Biochem. 2015;52(Pt 4):428-33.

10. Mashige F, Matsushima Y, Kanazawa H, Sakuma I, Takai N, Bessho F, Ohkubo A. Acidic catecholamine metabolites and 5-hydroxyindoleacetic acid in urine: the influence of diet. Ann. Clin. Biochem. 1996;33 ( Pt 1):43-9.

11. Perry TL, Hansen S, Hestrin M, MacIntyre L. Exogenous urinary amines of plant origin. Clin. Chim. Acta. 1965;11(1):24-34.

12. Nieman DC, Gillitt ND, Sha W, Esposito D, Ramamoorthy S. Metabolic recovery from heavy exertion following banana compared to sugar beverage or water only ingestion: A randomized, crossover trial. PLoS One. 2018;13(3).

13. Nieman DC, Gillitt ND, Sha W, Meaney MP, John C, Pappan KL, Kinchen JM. Metabolomics-Based Analysis of Banana and Pear Ingestion on Exercise Performance and Recovery. J. Proteome Res. 2015;14(12):5367-77.

14. Vázquez-Manjarrez N, Weinert CH, Ulaszewska M, Pétéra M, Micheau P, Durand S, Pujos-Guillot E, Mack CI, Egert B, Mattivi F, et al. Discovery and validation of banana intake biomarkers using untargeted metabolomics. 2019.

15. Wang Y, Gapstur SM, Carter BD, Hartman TJ, Stevens VL, Gaudet MM, McCullough ML. Untargeted Metabolomics Identifies Novel Potential Biomarkers of Habitual Food Intake in a Cross-Sectional Study of Postmenopausal Women. J. Nutr. 2018;148(6):932-43.

16. Helander A, Beck O, Boysen L. 5-hydroxytyptophol conjugation in man-influence of alcohol consumption and altered serotonin turnover. Life Sciences. 1995;56(18):1529-34.

17. Mathew S, Halama A, Kader SA, Choe M, Mohney RP, Malek JA, Suhre K. Metabolic changes of the blood metabolome after a date fruit challenge. J. Funct. Foods. 2018;49:267-76.

18. Sae-Teaw M, Johns J, Johns NP, Subongkot S. Serum melatonin levels and antioxidant capacities after consumption of pineapple, orange, or banana by healthy male volunteers. J. Pineal Res. 2013;55(1):58-64.

19. Johns NP, Johns J, Porasuphatana S, Plaimee P, Sae-Teaw M. Dietary intake of melatonin from tropical fruit altered urinary excretion of 6-sulfatoxymelatonin in healthy volunteers. J. Agric. Food Chem. 2013;61(4):913-9.

20. Feldman JM, Lee EM, Castleberry CA. Catecholamine and serotonin content of foods-Effect on urinary excretion of homovanillic and 5-hydroxyindoleacetic acid. J. Am. Diet. Assoc. 1987;87(8):1031-5.

21. Pallister T, Jennings A, Mohney RP, Yarand D, Mangino M, Cassidy A, MacGregor A, Spector TD, Menni C. Characterizing Blood Metabolomics Profiles Associated with Self-Reported Food Intakes in Female Twins. PLoS One. 2016;11(6):e0158568.

22. Quiros-Sauceda AE, Chen CO, Blumberg JB, Astiazaran-Garcia H, Wall-Medrano A, Gonzalez-Aguilar GA. Processing 'Ataulfo' Mango into Juice Preserves the Bioavailability and Antioxidant Capacity of Its Phenolic Compounds. Nutrients. 2017;9(10).

23. Barnes RC, Krenek KA, Meibohm B, Mertens-Talcott SU, Talcott ST. Urinary metabolites from mango (Mangifera indica L. cv. Keitt) galloyl derivatives and in vitro hydrolysis of gallotannins in physiological conditions. Mol. Nutr. Food Res. 2016;60(3):542-50.

24. Gouado I, Schweigert FJ, Ejoh RA, Tchouanguep MF, Camp JV. Systemic levels of carotenoids from mangoes and papaya consumed in three forms (juice, fresh and dry slice). Eur. J. Clin. Nutr. 2007;61(10):1180-8.

25. Garcia-Villalba R, Carlos Espin J, Tomas-Barberan FA. Chromatographic and spectroscopic characterization of urolithins for their determination in biological samples after the intake of foods containing ellagitannins and ellagic acid. J. Chromatogr. A. 2016;1428:162-75.

26. Tomas-Barberan F, Gonzalez-Sarrias A, Garcia-Villalba R, Romo-Vaquero M, Beltran D, Selma V, Carlos Espin J. Stratification by gut microbiota metabotypes can explain differences in response to polyphenol dietary interventions. Abstracts of Papers of the American Chemical Society. 2018;256.

27. Tomás-Barberán FA, García-Villalba R, González-Sarrías A, Selma MV, Espín JC. Ellagic Acid Metabolism by Human Gut Microbiota: Consistent Observation of Three Urolithin Phenotypes in Intervention Trials, Independent of Food Source, Age, and Health Status. J. Agric. Food Chem. 2014;62(28):6535-8.

28. Roberts KM, Grainger EM, Thomas-Ahner JM, Hinton A, Gu J, Riedl KM, Vodovotz Y, Abaza R, Schwartz SJ, Clinton SK. Application of a low polyphenol or low ellagitannin dietary intervention and its impact on ellagitannin metabolism in men. Mol. Nutr. Food Res. 2017;61(3).

29. Yang J, Lee R, Henning SM, Thames G, Hsu M, ManLam H, Heber D, Li Z. Soy protein isolate does not affect ellagitannin bioavailability and urolithin formation when mixed with pomegranate juice in humans. Food Chem. 2016;194:1300-3.

30. Mertens-Talcott SU, Jilma-Stohlawetz P, Rios J, Hingorani L, Derendorf H. Absorption, metabolism, and antioxidant effects of pomegranate (Punica granatum l.) polyphenols after ingestion of a standardized extract in healthy human volunteers. J Agric Food Chem. 2006;54(23):8956-61.

31. Romo-Vaquero M, García-Villalba R, González-Sarrías A, Beltrán D, Tomás-Barberán FA, Espín JC, Selma MV. Interindividual variability in the human metabolism of ellagic acid: Contribution of Gordonibacter to urolithin production. Journal of Functional Foods. 2015;17:785-91.

32. Gonzalez-Sarrias A, Garcia-Villalba R, Romo-Vaquero M, Alasalvar C, Orem A, Zafrilla P, Tomas-Barberan FA, Selma MV, Espin JC. Clustering according to urolithin metabotype explains the interindividual variability in the improvement of cardiovascular risk biomarkers in overweight-obese individuals consuming pomegranate: A randomized clinical trial. Mol. Nutr. Food Res. 2017;61(5).

33. Seeram NP, Henning SM, Zhang Y, Suchard M, Li Z, Heber D. Pomegranate juice ellagitannin metabolites are present in human plasma and some persist in urine for up to 48 hours. The Journal of nutrition. 2006;136(10):2481-5.

34. González-Sarrías A, García-Villalba R, Núñez-Sánchez MA, Tomé-Carneiro J, Zafrilla P, Mulero J, Tomás-Barberán FA, Espín JC. Identifying the limits for ellagic acid bioavailability: A crossover pharmacokinetic study in healthy volunteers after consumption of pomegranate extracts. Journal of Functional Foods. 2015;19:225-35.

35. Seeram NP, Zhang Y, McKeever R, Henning SM, Lee RP, Suchard MA, Li Z, Chen S, Thames G, Zerlin A, et al. Pomegranate juice and extracts provide similar levels of plasma and urinary ellagitannin metabolites in human subjects. J Med Food. 2008;11(2):390-4.

36. Nunez-Sanchez MA, Garcia-Villalba R, Monedero-Saiz T, Garcia-Talavera NV, Gomez-Sanchez MB, Sanchez-Alvarez C, Garcia-Albert AM, Rodriguez-Gil FJ, Ruiz-Marin M, Pastor-Quirante FA, et al. Targeted metabolic profiling of pomegranate polyphenols and urolithins in plasma, urine and colon tissues from colorectal cancer patients. Mol. Nutr. Food Res. 2014;58(6):1199-211.

37. Cerda B, Espin JC, Parra S, Martinez P, Tomas-Barberan FA. The potent in vitro antioxidant ellagitannins from pomegranate juice are metabolised into bioavailable but poor antioxidant hydroxy-6H-dibenzopyran-6-one derivatives by the colonic microflora of healthy humans. Eur J Nutr. 2004;43(4):205-20.

38. Mandel H, Levy N, Izkovitch S, Korman SH. Elevated plasma citrulline and arginine due to consumption of Citrullus vulgaris (watermelon). J. Inherit. Metab. Dis. 2005;28(4):467-72.

39. Bailey SJ, Blackwell JR, Williams E, Vanhatalo A, Wylie LJ, Winyard PG, Jones AM. Two weeks of watermelon juice supplementation improves nitric oxide bioavailability but not endurance exercise performance in humans. Nitric Oxide. 2016;59:10-20.

40. Collins JK, Wu G, Perkins-Veazie P, Spears K, Claypool PL, Baker RA, Clevidence BA. Watermelon consumption increases plasma arginine concentrations in adults. Nutrition. 2007;23(3):261-6.

41. Shanely RA, Nieman DC, Perkins-Veazie P, Henson DA, Meaney MP, Knab AM, Cialdell-Kam L. Comparison of Watermelon and Carbohydrate Beverage on Exercise-Induced Alterations in Systemic Inflammation, Immune Dysfunction, and Plasma Antioxidant Capacity. Nutrients. 2016;8(8).

42. Edwards AJ, Vinyard BT, Wiley ER, Brown ED, Collins JK, Perkins-Veazie P, Baker RA, Clevidence BA. Consumption of watermelon juice increases plasma concentrations of lycopene and beta-carotene in humans. J. Nutr. 2003;133(4):1043-50.

43. Schweiggert RM, Kopec RE, Villalobos-Gutierrez MG, Högel J, Quesada S, Esquivel P, Schwartz SJ, Carle R. Carotenoids are more bioavailable from papaya than from tomato and carrot in humans: a randomised cross-over study. The British journal of nutrition. 2014;111(3):490-8.

44. Irwig MS, El-Sohemy A, Baylin A, Rifai N, Campos H. Frequent intake of tropical fruits that are rich in beta-cryptoxanthin is associated with higher plasma beta-cryptoxanthin concentrations in Costa Rican adolescents. J. Nutr. 2002;132(10):3161-7.
