# Supplementary material for: Biomarkers of intake for tropical fruits
Source: Genes Nutr. 2020 Jun 19;15:11. doi: 10.1186/s12263-020-00670-4 (PMC7304196; doi:10.1186/s12263-020-00670-4)
Supplement: Supplementary file 3 — Additional file 3: Figure S1. Specific compounds found in different tropical fruits that may be further explored as putative BFIs in human studies. [file 12263_2020_670_MOESM3_ESM.pptx]

## Slide 1
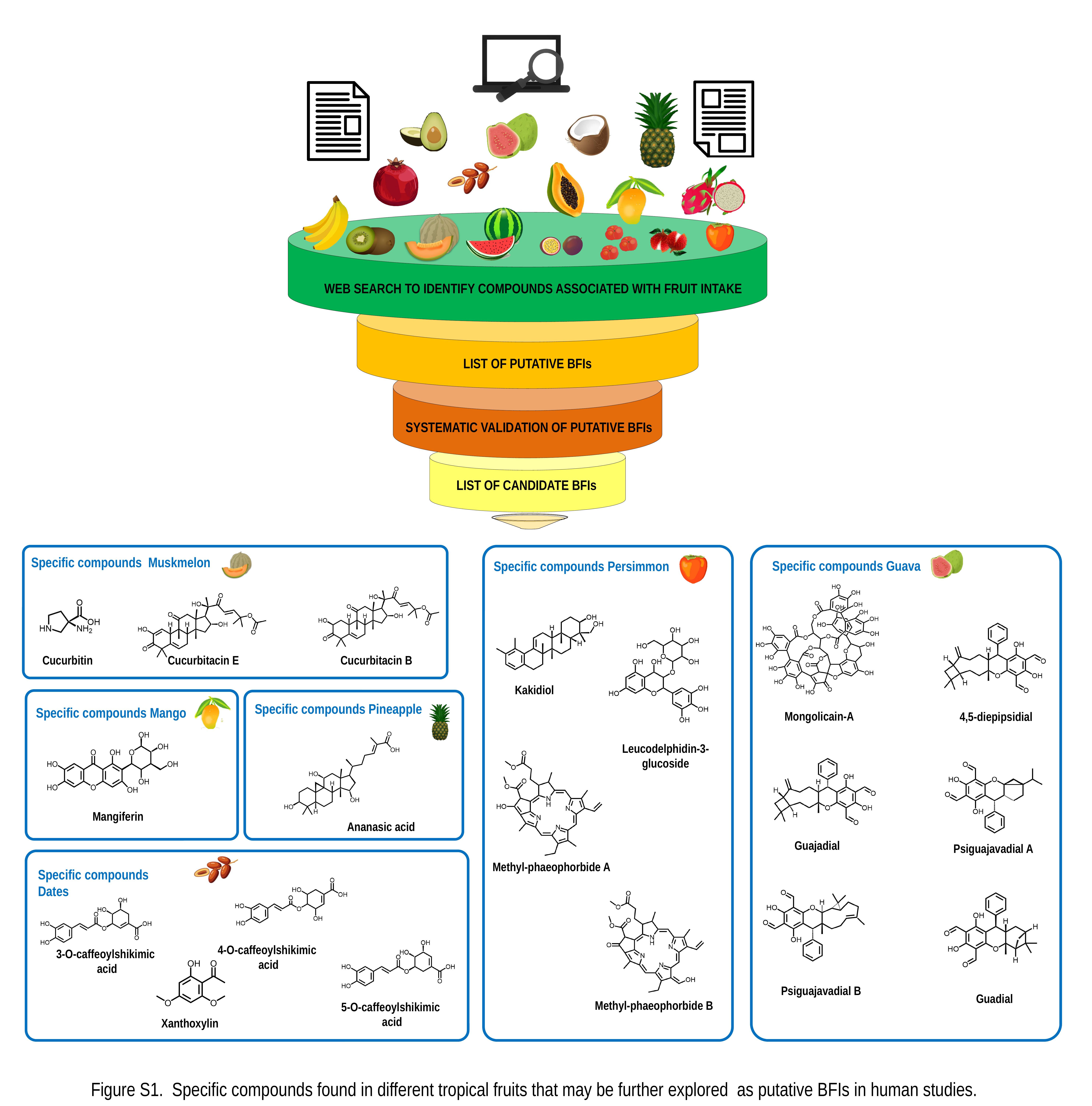

WEB SEARCH TO IDENTIFY COMPOUNDS ASSOCIATED WITH FRUIT INTAKE
LIST OF PUTATIVE BFIs
SYSTEMATIC VALIDATION OF PUTATIVE BFIs
LIST OF CANDIDATE BFIs
Specific compounds Muskmelon
Cucurbitin
Cucurbitacin E
Cucurbitacin B
Specific compounds Persimmon
Kakidiol
Leucodelphidin-3-glucoside
Methyl-phaeophorbide A
Methyl-phaeophorbide B
Specific compounds Guava
Mongolicain-A
4,5-diepipsidial
Guajadial
Psiguajavadial A
Psiguajavadial B
Guadial
Specific compounds Pineapple
Ananasic acid
Specific compounds Mango
Mangiferin
Specific compounds Dates
4-O-caffeoylshikimic
acid
3-O-caffeoylshikimic
 acid
5-O-caffeoylshikimic
acid
Xanthoxylin
Figure S1. Specific compounds found in different tropical fruits that may be further explored as putative BFIs in human studies.
